# Measuring eye vergence angle in extended reality

**DOI:** 10.1371/journal.pone.0333043

**Published:** 2025-11-25

**Authors:** Mohammed Safayet Arefin, John Edward Swan II, Russell Cohen Hoffing, Steven M. Thurman

**Affiliations:** 1 Department of Computer Science, Colorado State University, Fort Collins, Colorado, United States of America; 2 Department of Computer Science and Engineering, Mississippi State University, Starkville, Mississippi, United States of America; 3 US DEVCOM Army Research Laboratory, Los Angeles, California, United States of America; The Ohio State University, UNITED STATES OF AMERICA

## Abstract

Recently, extended reality (XR) displays, including augmented reality (AR) and virtual reality (VR), have integrated eye tracking capabilities, which could enable novel ways of interacting with XR content. In natural settings, eye vergence angle (EVA) changes constantly, based on the distance of fixated objects. Here, we measured EVA for eye fixations on real and virtual target objects in three different environments: real objects in the real world (real), virtual objects in the real world (AR), and virtual objects in a virtual world (VR). In a repeated measures design with 13 participants, EVA was measured while participants fixated on targets at varying distances. As expected, the results showed a significant main effect of target depth such that increasing EVA was associated with closer targets. However, there were consistent individual differences in baseline EVA. There was also a smaller but statistically significant main effect of environment (real, AR, VR) on EVA. Importantly, EVA was stable with respect to the starting depth of previously fixated targets and invariant to the direction (convergence vs. divergence) of vergence changes. In addition, EVA proved to be a more veridical depth estimate than verbal subjective depth judgments.

## 1 Introduction

Over the past several decades, Extended Reality (XR) technology has progressed rapidly, leading to increasingly capable and lower-cost head-mounted displays. These displays generally encompass Virtual Reality (VR), which immerses observers in a virtual world, and Augmented Reality (AR), in which observers see virtual objects superimposed on the real world. Due to the rapid development of this technology, many novel applications of XR have been developed in areas including surgery, training, maintenance, education, entertainment, and others [1,2]. Recently, to provide automated calibration and a better user experience, many such displays have included built-in eye tracking technology (e.g., Microsoft HoloLens 2, Magic Leap 2, HTV Vive Pro Eye, and others). In this research, we measured *Eye Vergence Angle* (EVA) from an eye tracker and investigated how EVA varies in real, AR, and VR environments for real and virtual objects placed at different depth locations from the observer.

**Fig 1:**
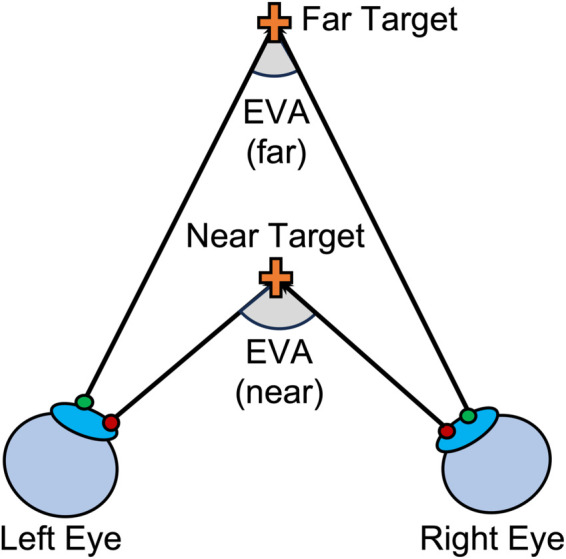
The geometry of the Eye Vergence Angle (EVA) while fixating on a near and far target. A closer target is associated with a larger EVA, and a farther target is associated with a smaller EVA.

**Eye vergence angle:** When fixating objects at different depths, the eyes undergo vergence eye movements and rotate horizontally [3]. The visual axes form the eye vergence angle (EVA) (Fig 1). Vergence eye movements can occur in two possible directions. When eye gaze shifts from fixating a far target to a near target, the rectus muscles rotate both eyes inward, resulting in *convergence*, and when eye gaze shifts from fixating on a near to a far target, the rectus muscles rotate both eyes outward, resulting in *divergence* (Fig 1). Therefore, the value of EVA varies when fixating objects at different depths, and the geometry of binocular vision suggests that EVA is smaller while fixating on a far object and larger while fixating on a near object. When fixating objects close to infinity, the visual axes are close to parallel, and EVA approaches zero.

**Goal and approach:** For this work, our initial motivation was to replicate and extend Arefin *et al*. [4], which measured EVA when fixating virtual targets in a VR environment. In particular, our goal was to compare these measurements against a control condition of carefully calibrated real targets in the real world. However, in a head mounted VR display it is not possible to see the real world, and therefore it is difficult to calibrate virtual targets to real world targets [5]. We solved this problem by using an optical see-through AR display (a Microsoft HoloLens 2), because in such a display, the real world can be seen, and therefore the alignment between virtual and real objects can be measured and precisely calibrated. Then, by covering the front of the display with an opaque cover, the HoloLens 2 can become a VR display in which only virtual content is perceived. Therefore, this experiment used targets that were carefully matched in position and disparity depth to measure EVA in real, AR, and VR environments. All measurements were collected in a single repeated measures experiment. To the best of our knowledge, this is the first experiment to measure EVA in response to carefully calibrated objects located at different depths in real, AR, and VR environments. In addition, subjective verbal reports of perceived depth were also collected, which allowed us to replicate previous findings of depth underestimation for XR virtual objects, and for direct comparison to EVA.

**Hypotheses:** In this experiment, we hypothesized that (**H1**) EVA would co-vary with the depth of fixated objects in all environments, such that fixations to nearer objects would be associated with a larger EVA and fixations to farther objects with a smaller EVA (as illustrated in Fig 1). However, due to the vergence-accommodation conflict and previous work suggesting perceptual underestimation of virtual objects, we also hypothesized that (**H2**) the functional mapping of EVA to depth in AR and VR environments would differ from the real world environment. In addition, when fixating on an object, we hypothesized that (**H3**) the mapping of EVA to object depth would not vary with the depth location of the previously fixated object. We termed this the *vergence stability hypothesis*. It predicts that, for example, when an observer fixates a target at 1.5 meters, the EVA should be the same regardless of whether the observer previously changed their gaze from .25 to 1.5 meters (divergence) or from 4 to 1.5 meters (convergence). Testing this hypothesis requires examining whether the relationship between EVA and object depth is stable across a range of convergence and divergence eye movements. Lastly, we compared EVA measurements with subjective judgments of depth perception collected from verbal reports of depth from the same set of real, AR and VR targets. Although the finding that the depth of virtual objects is generally underestimated has been widely reported, we hypothesized that (**H4**) EVA would provide a more veridical estimate of depth for virtual objects. We reasoned that objective measurements of EVA would be less susceptible to cognitive or perceptual biases and would reflect the reflexive oculomotor response to real and virtual objects at different depth locations. Given the novelty of measuring EVA in XR environments, H4 did not predict a particular direction (i.e., overestimation or underestimation) of depth-related errors associated with EVA. In summary, the hypotheses that motivated this experiment are as follows:

- **H1**: EVA will covary with fixated target depth.
- **H2**: EVA will differ according to whether the targets are in a real, AR, or VR environment.
- **H3**: When fixating a target, the EVA will be stable, regardless of the focal switching depth or vergence direction of the eye movement (the vergence stability hypothesis).
- **H4**: EVA will provide a more veridical depth estimate than subjective depth judgments, and subjective judgments will be underestimated.

## 2 Background and related work

### 2.1 Accommodation and vergence

In XR environments, information can be presented at different depths from the observer’s eye position. The accuracy and precision of depth perception of objects depends on the user’s eye movements (e.g., saccadic, fixation, vergence, smooth pursuit, etc.) and the presence of monocular and binocular depth cues (e.g., accommodation, vergence, relative size, blur, binocular disparity, etc.) in the perceived scene [6–8]. When fixating an object at a particular depth, the eyes’ ciliary muscles change the eye lens shape to see information in sharp focus. Known as *accommodation*, this change seeks to minimize *retinal blur* [9]. In addition, in binocular vision, fixating an object at a particular depth requires simultaneous *vergence eye movements*, controlled by the eye’s rectus muscles. Eye vergence is stimulated primarily by *fusional vergence (disparity vergence)*: the amount of eye rotation needed to avoid double vision. Vergence eye movements bring two eye images of the viewed object to the center of the fovea [10]. Furthermore, accommodation and vergence are coupled by the *accommodation-vergence reflex* [8,11,12]. This reflex leads any changes in accommodation to cause changes in vergence (*accommodative vergence*), and any changes in vergence to cause changes in accommodation (*vergence accommodation*). Both accommodation and vergence are also linked to pupil diameter, which controls the eye’s focal depth of field; the three responses all drive and are driven by each other [13].

Under real-world binocular viewing, accommodation and vergence co-vary with the depth of the fixated object. However, when using a stereo display with a single or fixed focal plane, the human visual system can override the accommodation-vergence reflex [7,8]. Because the optical depth of the display plane sets the accommodative demand, viewers using head-mounted XR displays must binocularly fuse virtual objects at depths that may differ from the display’s optical depth, with a vergence demand different from the appropriate accommodative demand [14–16]. This inconsistency is called the *accommodation-vergence* mismatch problem. This is a longstanding issue for commercial XR displays, and can cause many perceptual problems, including misperception of depth, eye strain, double-vision, and others [11,16,17].

### 2.2 Measuring Eye Vergence Angle (EVA)

In studies of the human visual system, researchers have long used eye tracking devices to measure EVA. Typically, EVA has been calculated from each eye’s line of sight. Primarily, EVA has been collected from fixating real objects, using controlled setups that sometimes have restricted head movements [7,18–23]. In the XR research community, only a few prior studies have measured EVA while fixating on virtual objects, using both custom-mounted eye trackers and newer displays with built-in eye trackers. These studies have tracked perceptual depth in VR [4,24], developed a vergence-controlled gaze interaction method [25], and estimated the gaze depth for varifocal displays [26]. Duchowski *et al*. [27,28] directly measured and compared the gaze depth between real and virtual objects. They used a commercial binocular eye tracker, a custom-built VR display, and real objects. Interestingly, they observed that the vergence error was relatively small in the virtual environment compared to the physical environment. Later, Iskander *et al*. [29] compared the vergence angle between the ideal and VR conditions, where the EVA for the ideal condition was calculated through a biomechanical simulation using inverse kinematics, and the EVA for the VR condition was computed from the gaze vectors provided by an eye tracker. They found that the EVA of the eye tracker exhibited more variability and higher values than the simulated ideal condition, which they attributed to the vergence-accommodation conflict in the VR headset.

### 2.3 Depth perception in XR

When measuring perceived depth in XR environments, researchers have used both cognitive and perception-action based depth judgment tasks, including verbal reporting [30], perceptual matching [31], blind reaching [31], blind walking [32], and triangulated walking [33,34]. Compared to the depth of real objects in the real world, this work has generally found that the depth of virtual objects is misperceived. In AR, both depth underestimation [30,31,35] and overestimation [36] have been reported. In VR, most previous research has found depth underestimation of VR objects compared to real objects [32,37,38], although in modern VR displays the amount of underestimation has declined [39,40]. While this research has extensively explored the cognitive and perceptual phenomena behind XR depth perception, we have not found a previous study that measures EVA while fixating on objects at different depths in real, AR, and VR environments. Therefore, the effect of the environment on EVA has not previously been measured.

### 2.4 Gaze-adaptive XR user interfaces

Initially, eye trackers were exclusively used to study perception, cognitive processes, and reading and related information processing tasks [41]. However, beginning in the 1980s, eye trackers began to be tested as inputs to human-computer interaction techniques [42]. Initial work involved desktop computers and atomic user interaction tasks such as object selection, object movement, scrolling, and menu selection [43]. Within desktop and tablet-based computing, eye tracking has been used for increasingly sophisticated tasks [42,44,45]. Within user interface development, eye tracking can sometimes allow inferring a user’s intent, resulting in more capable and adaptive interaction techniques [42,45,46].

The motivation of this work is the increasing availability of eye tracking in head-worn XR devices. While eye tracking was initially deployed in head-worn displays to improve calibration [47], its ability to improve interaction has received attention from both the computer-human interaction and mixed reality research communities [48]. A recent survey article by Plopski *et al*. [46] covering gaze interaction and eye tracking in head-worn XR finds 215 papers spanning the years 1985 to 2020, with the majority published after 2013. These cover interacting with virtual content, designing interfaces that adapt content based on eye gaze behavior, and using eye gaze to improve collaboration in XR. The paper concludes that recent advances in gaze interaction are largely driven by advances in eye tracking hardware and gaze estimation algorithms. It also finds that the great majority of the surveyed work largely adapts solutions from 2D interfaces, and do not fully utilize the 3D structure of XR interaction space. As the first experiment to measure and compare eye vergence angle in real, AR, and VR environments, our work directly contributes to both of these conclusions.

## 3 Method

### 3.1 Participants

Sixteen participants, 13 males and 3 females, were recruited from the Mississippi State University community. The Institutional Review Board (IRB) for the Protection of Human Subjects at Mississippi State University approved the study protocol (IRB 17-251) in accordance with the Declaration of Helsinki, and all participants provided written consent. This approval covers the collection, storage, and analysis of the data. The data is available according to the Data Availability Statement associated with this paper.

Participants for the experiment reported in the paper were recruited beginning in mid-November, 2022, and the data was collected between December 1, 2022 and December 9, 2022. The participants’ ages ranged from 19 to 57 years, with a mean age of 29.9. Among them, 14 were right-eye dominant, and two were left-eye dominant. There were no visual restrictions for participation; 8 participants used corrective eyeglasses, while the remainder reported normal uncorrected vision. Participants’ mean interpupillary distance, measured at infinity, was 64.8 mm. Each participant was compensated at a rate of $12 per hour. Based on the data processing and quality analyses discussed in the Section Data Pre-Processing (below), data from 13 of these 16 participants were included in the final analysis.

### 3.2 Apparatus and setup

**Fig 2:**
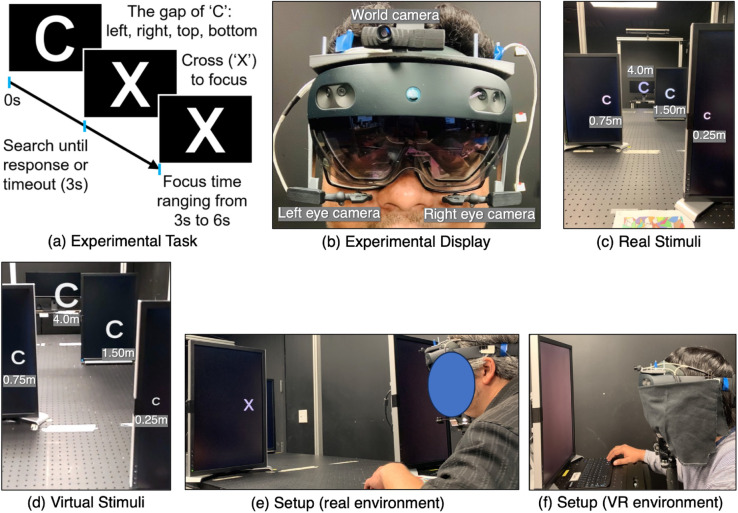
(a) The visual stimulus was a Landolt C resolving to a fixation cross, resulting in a four-alternative forced choice visual discrimination task. (b) Pupil Labs eye tracker custom mounted on a Microsoft HoloLens 2 display. (c) Real stimuli presented on monitors at four different depths. (d) Virtual stimuli presented at the same depth and position for the AR and VR environments. Note that the captured image does not reflect the actual perceptual experience of the user. (e) Participant performing the experiment in the real environment. (f) Participant performing the experiment in the VR environment.

The experiment was conducted in a 7.68 by 5.46 meter room. As the room was generally used for optical experiments, it was painted black and had no windows. The room contained a large 244 cm by 92 cm optical breadboard on which experimental equipment was positioned. During the experiment, participants were seated at one of the narrow ends of the optical breadboard (Fig 2e, 2f).

**Real visual stimulus:** The purpose of the real visual stimulus was to provide a fixation target that exists on a physical object in the real environment, positioned at a set of depths from the observer that could be objectively measured. The visual stimulus was a Landolt C with four directions, followed by a fixation cross (Fig 2a). The real visual stimulus was presented on four physical monitors positioned at four different depths relative to the participant’s eye location (Fig 2c). Three of the monitors (Dell UltraSharp U2211H) were identical, each with a display resolution of 1920×1080, 40.05 pixels per cm, and a diagonal size of 55 cm. The fourth monitor (Dell UltraSharp U2913WM) had a display resolution of 2560×1080, 29 pixels per cm, a diagonal size of 73.66 cm, and was placed on the top of a locked rolling cart. The four monitors were positioned in such a way that participants had a clear line of sight to the visual targets presented on each monitor. The size of the target stimulus at each distance was scaled to achieve a constant size in terms of degrees of visual angle relative to the observer.

**Virtual visual stimulus:** Throughout the experiment, participants wore a Microsoft HoloLens 2 binocular see-through head-mounted display (Fig 2b). According to the manufacturer, the HoloLens 2 has a 2K resolution, weighs 566g, and has a diagonal field of view of 52^°^. The participants wore the display when they observed the real visual stimulus (Fig 2c), but the display was turned off. When observing the AR visual stimulus, participants saw the virtual target objects positioned on the monitors, which were turned off (Fig 2d). When observing the VR visual stimulus (Fig 2f), the display was covered with a black cloth, completely blocking the view of the experimental room. Participants saw the virtual target objects floating in blackness. The cloth was carefully placed to not block the HoloLens tracking cameras.

**Stimulus placement:** In all three stimulus environments, real, AR, and VR, target objects were placed 0.25, 0.75, 1.5, and 4.0 meters from the observer (Fig 2c, 2d). The target object at 4.0 meters was observed perpendicularly to the observer’s midline. Stimuli at other distances were positioned in such a way as to require head rotations of 5.71^°^ right (1.5 meters), 7.60^°^ left (0.75 meters), and 25^°^ right (0.25 meters) to shift the fixation from perpendicularly facing the far distance to perpendicularly facing the new, closer distance. However, the experimenters observed that the actual amount of head rotation varied slightly between different participants. Both real and virtual stimuli were rendered in white, and each stimulus had a constant visual angle size of 3.87^°^. This ensured that the size of the stimuli did not vary with distance, similar to previous experiments [6,49].

When using optical see-through AR displays, placing virtual objects at precise locations in the real world has historically been challenging. Khan *et al*. [50–52] developed an optical tracking algorithm that operates through the front-facing camera of the HoloLens to establish the virtual location of a real-world fiducial mark. They showed that with this approach, the perceived alignment error between real and virtual object locations could be as low as a few millimeters. Therefore, we adapted the method from Khan *et al*. [50,52] to place a virtual object at a highly precise location in the real world environment.

The closest target object was 25 cm from the observer (Fig 2c, 2d). While this distance was possible to display through the HoloLens 2, the Magic Leap 2, the other popular optical see-through AR display at the time we ran this experiment, could not show a virtual object closer than 35 cm from the observer [53]. Consequently, in our experiment all visual stimuli could be binocularly focused at all experimental distances.

**Eye tracking:** Calculating EVA requires an eye gaze ray (origin and direction) from both eyes. Although the Microsoft HoloLens 2 has eye tracking cameras for both eyes, when this experiment was conducted, Microsoft’s Mixed Reality Toolkit eye tracking API only provided a single eye gaze ray (later, the API did add the ability to obtain an eye gaze ray from both eyes). It was therefore not possible to know whether the right or left eye gaze ray was being returned, and it was not possible to compute EVA from the HoloLens 2 eye tracking cameras. Therefore, a Pupil Labs Pupil Core stereo eye tracking system was mounted on the HoloLens 2, using a custom-designed 3D printed scaffolding (Fig 2b). Each eye tracking camera was carefully placed to have a clear view of the eye and to not block the participant’s field of view. According to the manufacturer, the eye tracking system has an accuracy of .60^°^, a precision of .02^°^, a sampling rate of 200 Hz, and a resolution of 192×192.

**Computing platform:** The entire experiment was controlled by two separate Unity programs, a stand-alone Unity application (Unity version: 2021.3.4f1) for real targets, and another mixed reality Unity application (Unity version: 2020.2.2f1) for AR and VR targets. Both programs used Microsoft’s Mixed Reality Toolkit, and ran on a Windows 10 desktop (HP Z2 Tower G4 Workstation), with an Intel Core i-9 CPU running at 3.60 GHz with 64 GB of RAM. The eye tracking data was gathered using the Unity Pupil Labs eye tracking API. Offline eye movement data processing, analysis, and visualization were performed with Matlab (R2022a) and R (4.4.2). A numeric keyboard was used to gather the participant’s responses.

### 3.3 Experimental task

The experimental task aimed to obtain quantitative EVA measurements as participants shifted their gaze to fixate on stimuli at different depths. A four-alternative forced choice visual discrimination task (Landolt C) required participants to successfully shift their fixation from one target to the next. Spiegel and Erkelens [16] used a very similar Landolt C task to induce accommodation to specific depths in augmented reality. A trial began when a single capital letter *C* in a sans-serif font (Arial) appeared at a certain depth. For each trial, the gap of the *C* was randomly chosen to be left, right, top, or bottom. The participants’ task was to determine the gap direction. After the participant’s response, or after the maximum allowed time of 3 seconds, the letter *C* changed to *X* (Fig 2a). Participants were instructed to focus on the center of the *X* for an intertrial interval that lasted for a randomly chosen time of 3 to 6 seconds. When this interval was completed, the trial ended. Then a new letter *C* appeared at a new depth location, beginning the next trial. Participants were instructed to immediately shift their gaze to the new target letter.

### 3.4 Variables and design

The experiment examined two different independent variables: *environment* and *depth*. Three levels of the *environment* were considered: *real*, *AR*, and *VR*. The distance from the participant’s eye position to the stimulus position was *depth*. Four levels were considered (Table 1): 0.25, 0.75, 1.5, and 4.0 meters (m), or 4.0, 1.33, 0.67, and 0.25 diopters (D), where diopters = 1/meters. In each experimental trial, participants made a *vergence eye movement* that changed their gaze from a target at the *start depth* to a target at the *end depth*, resulting in a particular *focal switching depth*. These depth changes resulted in 12 possible permutations; 6 were divergence eye movements, and 6 were convergence eye movements.

**Table 1:** The experiment placed targets at 4 different depths, resulting in 12 different vergence eye movements. Measurements are expressed in meters (m) and diopters (D).

Depth Pair	Start Depth		End Depth		Focal Switching Depth		Vergence Eye Movement
1	0.25m	(4.0D)	0.75m	(1.33D)	0.5m	(2.67D)	Diverge
2	0.25m	(4.0D)	1.50m	(0.67D)	1.25m	(3.33D)	Diverge
3	0.25m	(4.0D)	4.0m	(0.25D)	3.75m	(3.75D)	Diverge
4	0.75m	(1.33D)	1.50m	(0.67D)	0.75m	(0.66D)	Diverge
5	0.75m	(1.33D)	4.0m	(0.25D)	3.25m	(1.1D)	Diverge
6	1.50m	(0.67D)	4.0m	(0.25D)	2.50m	(0.42D)	Diverge
7	4.0m	(0.25D)	1.50m	(0.67D)	2.50m	(0.42D)	Converge
8	4.0m	(0.25D)	0.75m	(1.33D)	3.25m	(1.1D)	Converge
9	4.0m	(0.25D)	0.25m	(4.0D)	3.75m	(3.75D)	Converge
10	1.50m	(0.67D)	0.75m	(1.33D)	0.75m	(0.66D)	Converge
11	1.50m	(0.67D)	0.25m	(4.0D)	1.25m	(3.33D)	Converge
12	0.75m	(1.33D)	0.25m	(4.0D)	0.5m	(2.67D)	Converge

A within-subjects, repeated-measures design was employed. Each combination of environment and depth pair was repeated six times. Therefore, each participant observed 3 (*environment*) × 12 (*depth pair*) = 36 conditions, where each condition was repeated 6 times, for a total of 216 trials per participant in a complete experimental session. Between participants, the presentation order of environment was randomly chosen from either *real-AR-VR*, *AR-VR-real*, or *VR-AR-real*. The real condition was either shown first or last, because it required switching on and calibrating the display, or switching it off. Within each participant, the presentation order of *depth pair* × *repetition* was randomly permuted, with the restriction that there was no back-to-back repetition of the same depth pair. At the beginning of the experiment, the first start depth was randomly chosen. For each subsequent trial, the end depth of trial *i* was the start depth of trial *i* + 1. For each trial, the participant’s continuous real-time left and right eye movement data (gaze direction vector, gaze origin vector, gaze normal vector, eye center vector, gaze data confidence level), the response of the Landolt C task (left, right, bottom, top), and the button press timestamp were collected.

In addition, for each participant, 3 verbal distance estimates were collected for each environment (real, AR, VR) and distance (0.25, 0.75, 1.5, 4.0 meters).

### 3.5 Procedure

**Step 1: Preliminary tasks:** A participant started the experiment by signing a consent form and filling out a general experimental questionnaire. Next, the participant’s interpupillary distance was measured at infinity with a commercial pupilometer, and Miles’s test [54] was employed to determine the dominant eye. A detailed description of the experiment was then provided. During this process, the experimenter presented examples of stimuli on paper so that the participants could familiarize themselves with the stimuli and the task. Next, the experimenter helped the participant put on the HoloLens 2, seeking a proper fit that reduced unwanted slippage.
**Step 2: HoloLens 2 calibration:** Upon switching on the HoloLens 2, the participant performed two separate calibration procedures. First, the participant performed the standard HoloLens 2 calibration procedure. This ran the proprietary HoloLens 2 calibration software, which used the built-in HoloLens 2 eye trackers. We can assume that the calibration measured the participant’s interpupillary distance and other features of the eye position relative to the display’s tracking coordinate frame [47].
**Step 3: Eye tracker calibration:** Next, the participant performed a procedure to calibrate the Pupil Labs Pupil Core eye tracker. The participants sat on a chair within arm’s length distance (∼60 cm) from the Pupil Labs monitor. The experimenter then adjusted the eye tracking cameras so the participant’s pupils and eyeballs were clearly visible and checked the pupil labs’ pupil capture software to ensure that, for both eyes, the pupil capture model was operating well. Then, the calibration procedure was run. Five circular markers were displayed: four markers at the four corners and one at the center. Participants were instructed to follow and focus on the center of the calibration marker with minimal head movement.
**Step 4: Participant positioning:** After properly performing the display and eye tracker calibrations, participants sat on a tall height-adjustable chair, placed their chin on a chin rest, and placed their right hand on the numeric keyboard. The chair height was adjusted so that participants of different heights could sit comfortably and all targets were at the same height as the eyes. The chin rest minimized head movement during the experiment.
**Step 5: Virtual-to-real calibration:** For AR and VR environments, participants next performed a third calibration procedure. This calibration, adopted from Khan *et al*. [50–52], allowed the HoloLens 2 to precisely position virtual objects in the real world. Participants were instructed to look at a printed fiducial marker that was attached to the optical workbench (Fig 2c). A machine vision tracking algorithm (Vuforia, PTC Inc.) running through the HoloLens forward-facing camera recognized the marker, and aligned the HoloLens virtual coordinate system with respect to the marker’s location. The HoloLens then rendered a virtual cube. Participants reported whether the virtual cube appeared to be located in the middle of the fiducial marker, and properly oriented. If so, the virtual coordinate system was properly calibrated. If not, the tracking system was restarted, and the procedure was repeated.
**Step 6: Verbal depth judgments:** Next, participants were shown the Landolt C target object, with the gap located to the right, at all four distances at the same time (Fig 2c). Participants were asked to verbally estimate the distance to each target, starting from the farthest and moving closer. Participants could use their desired units (feet, meters, inches). The experimenter encouraged the participant to modify their estimate as desired, and continued prompting until the participant had provided 3 estimates of each distance. Many, but not all, participants chose to give the same value 3 times. The experimenter recorded these verbal responses on paper.
**Step 7: Experimental trials:** The Landolt C trials for the current environment were then presented.
**Step 8: Remaining environments:** After collecting verbal depth judgments and experimental trial data for an environment (real, AR, or VR), steps 6 and 7 were repeated for the subsequent environments. The display was switched off for the real environment, and on for the AR and VR environments. When the display was first switched on, the calibration steps 2, 3, 4, and 5 were performed. When switching between AR and VR, the cloth was either attached to or removed from the display (Fig 2f). These procedures provided participants with a break between environments.
**Step 9: Final tasks:** After the experiment, an informal interview session was performed to collect additional thoughts, impressions, and insights. The participant was then compensated. The experiment lasted approximately one hour.

### 3.6 Data pre-processing

Before calculating EVA, we preprocessed the eye tracker data to omit blinks and noise artifacts associated with physiologically unrealistic data points. For omitted data points, we simply replaced them with *not a number* (NaN) in Matlab to preserve the full temporal structure of the raw data while ensuring that they would not be incorporated into the overall mean estimates.

We first preprocessed the collected eye tracker data by using the confidence value of each data point. According to the Pupil Labs eye tracker API, confidence values ranged continuously from 0 to 1, where 0 means the pupil was not detected at all, while 1 means the pupil was detected with high reliability. Data points with low confidence (<.75) were replaced with NaN values. Next, we performed velocity-based data filtering on vergence data to discard unrealistic physiological EVA values. Under this criteria, data points with very large velocities $\left(\text{EVA}>5000^{\circ /\text{second}}\right)$ were replaced with NaN values. Then, data points ≥2.5 standard deviations from the mean were replaced with NaN values. When applied to our data, these steps resulted in excluding an average, over all participants, of 16.72% of the data points for *real* (min participant: 4.19%, max participant: 47.20%); 14.58% for *AR* (min: 2.72%, max: 43.52%); and 18.54% for *VR* (min: 3.65%, max: 27.08%).

After obtaining the preprocessed eye-gaze data values from the previous step, we checked trial-by-trial validity. A trial with >50% valid eye-gaze data samples was considered valid. Based on this criteria, a total of 842 valid real trials (90.00% of the real trials), 893 valid AR trials (95.41% of the AR trials), and 879 valid VR trials (93.91% of the VR trials) were included.

The study considered 4 target distances that created 12 distinct depth pairs (Table 1). Each depth pair was repeated 6 times per participant per environment. Depth pairs that contained ≥3 valid trials out of 6 were considered valid. In addition, each participant experienced each environment. Environmental conditions with ≥6 valid depth pairs out of 12 were considered valid. A participant was considered valid if the participant’s data had three valid environmental conditions. According to this criteria, the data from two participants were discarded from the analysis. Another participant’s data was excluded due to eye tracker calibration issues. Therefore, 13 out of 16 participants were included in the analysis, resulting in 2489 EVA measurements.

**Fig 3:**
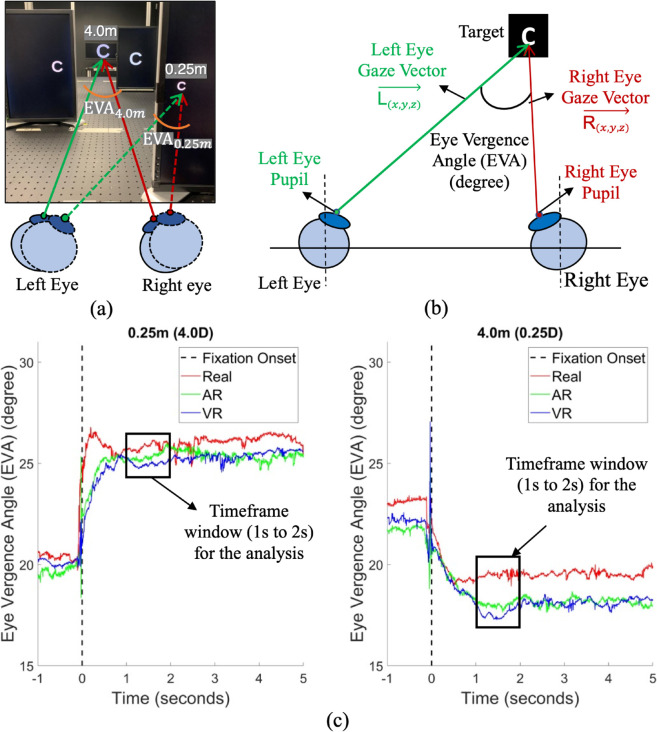
Eye Vergence Angle (EVA) calculation and an example of unprocessed eye tracking data. (a) The participant needs to rotate the head and eyes in different amounts to focus on objects at different distances, such as 0.25 m and 4.0 m (b). EVA is calculated from the 3D gaze direction vectors of the left and right eye. (c) An example of unprocessed eye tracking data at 0.25 m (4.0 D) and 4.0 m (0.25 D) for a participant. The vertical dotted line at 0 seconds shows fixation onset, which occurs approximately 250 ms after stimulus onset, and before the participant pressed the button. This example shows the difference in EVA values for the experiment’s two extreme depths (near and far) in the real, AR, and VR environments. For EVA analysis, a time window between 1 and 2 seconds after the fixation onset was used.

For each trial, we sought the first fixation that occurred <250 ms after stimulus onset, but before the button response was pressed. The analysis then focused on a time window of 1 to 2 seconds after fixation onset (Fig 3c). This ensured that the EVA had enough time to stabilize at the new target depth. We chose the 1 to 2 second window for the EVA analysis based on the timing of the perceptual and oculomotor system to perceive a target and make a saccadic eye movement to the correct spatial location (~250 ms), and the vergence eye movement to allow sharp focus on the stimulus (200–300 ms) [16,20,55,56].

The eye tracking data and Matlab code used for the processing described in this section are available according to the Data Availability Statement associated with this paper.

### 3.7 EVA calculation

When calculating EVA, we assumed unrestricted head and eye movements. Given that the 3D gaze direction vectors from the left and right eyes came from head-mounted eye trackers, we could assume that the 3D gaze vectors included head rotations, vergence eye movements, and interpupillary distance [26]. However, our experimental target positions did not meet the assumptions of the standard triangulation method because targets were not positioned perpendicularly to the eye position. Also, in our experiment, participants were free to rotate their heads to view the targets frontally. Previously, for experiments with real target objects, many researchers calculated EVA from 3D gaze direction vectors and vector intersection models [18,21]. Further, for experiments with virtual target objects seen in AR and VR environments, previous researchers also calculated EVA from 3D gaze vectors [4,25,26], and found that the vector intersection model was appropriate when eye movements were free. Therefore, we calculated EVA from the angle formed by the left and right eye 3D gaze vectors (Fig 3b). The 3D gaze direction vectors (*L*_(*x*,*y*,*z*)_ and *R*_(*x*,*y*,*z*)_) were projected on a plane, where *x* is the horizontal, *y* the vertical, and *z* the optical (depth) axes in the pupil labs 3D camera space coordinate system. Then, EVA was calculated as

$$\text{EVA}=\arccos\left(\frac{L_{\left(x,y,z\right)}\cdot R_{\left(x,y,z\right)}}{\left|L_{\left(x,y,z\right)}||R_{\left(x,y,z\right)}\right|}\right).$$ (1)

## 4 Results

As discussed above, the Landolt C task required a four-alternative forced-choice visual discrimination. A high score on this task demonstrated that participants attended to the task and focused at the correct depth. Behavioral results showed that participants’ mean accuracy was very high. The grand mean across all subjects and environmental conditions was 98%, with a standard deviation of 2.5% and a range of 90–100%. This indicates that all 13 participants paid attention and focused on the target at the presented depth location in nearly every trial.

Results were analyzed using *linear mixed-effects models* [57], where *participant* was the random effect, and *eye movement end depth* and *environment* (real, AR, or VR) were fixed effects. For each hypothesis, models were developed using a *step-down model-building approach* [58]. This approach begins by developing a *maximal model*, where each predictor includes all main effects and interactions. This maximal model is then subjected to a stepwise refinement algorithm, where terms are dropped with the goals of (1) simplifying the model while (2) not significantly reducing the model fit. The result is a *fitted model*. Then, to assess the fitted model, *R*^2^ terms are calculated. While for general mixed-effects models there is not a consensus for how to calculate *R*^2^ [59], for the simple linear mixed-effects models used here *R*^2^ can be calculated, and retains its standard meaning of the percentage of variance explained [60]. All of this constitutes statistical evidence that the fitted model is the most appropriate model to explain the results. We also considered whether the fitted model suggested an explanation that aligns with knowledge of eye movement behavior.

Data files and R code used for the analysis described in this section are available according to the Data Availability Statement associated with this paper.

### 4.1 End Depth (H1) and Environment (H2)

**Fig 4:**
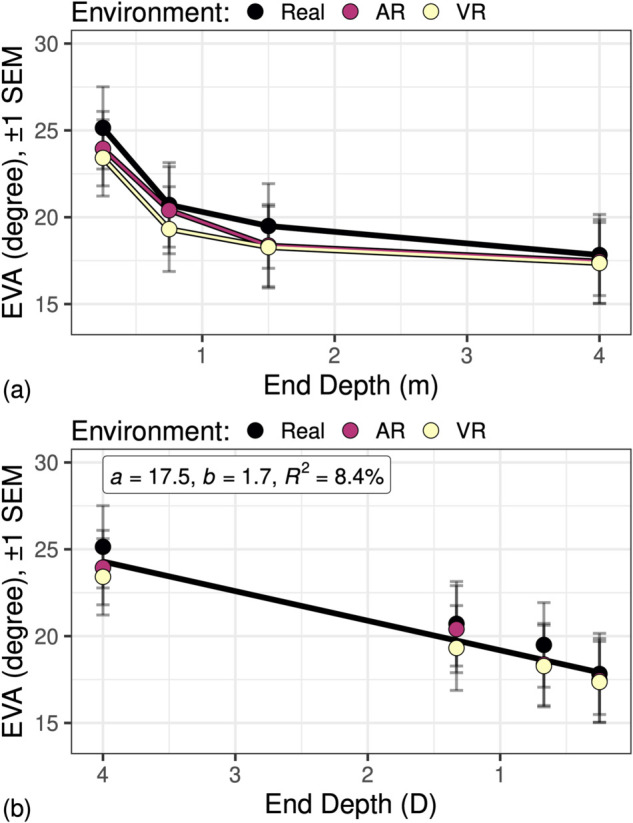
The Eye Vergence Angle (EVA) as predicted by (a) environment and eye movement end depth in meters, and (b) as predicted by eye movement end depth in diopters=1/meters. When expressed in diopters (D), the geometry of the eye vergence angle (Fig 1) results in a linear relationship between EVA and eye movement end depth. This can be seen in (b), where a linear model is fitted to the data, with the parameters of intercept (*a*), slope (*b*), and percentage of explained variation (*R*^2^).

We hypothesized that eye vergence angle (EVA) would covary with the depth of fixated target objects (Hypothesis H1), and that this covariation would also differ according to whether the environment included real, AR, or VR target objects (Hypothesis H2). Fig 4 shows the effects of environment and eye movement end depth on mean EVA. Fig 4a shows the depth in meters, while Fig 4b shows the same data with depth expressed in diopters=1/meters. While there is a non-linear relationship between depth and EVA due to the geometry of the eye vergence system (Fig 1), this relationship can be expressed as a linear relationship by converting depth to units of diopters (D). Our statistical modeling was therefore applied to data in which depth is expressed in diopters.

**Fig 5:**
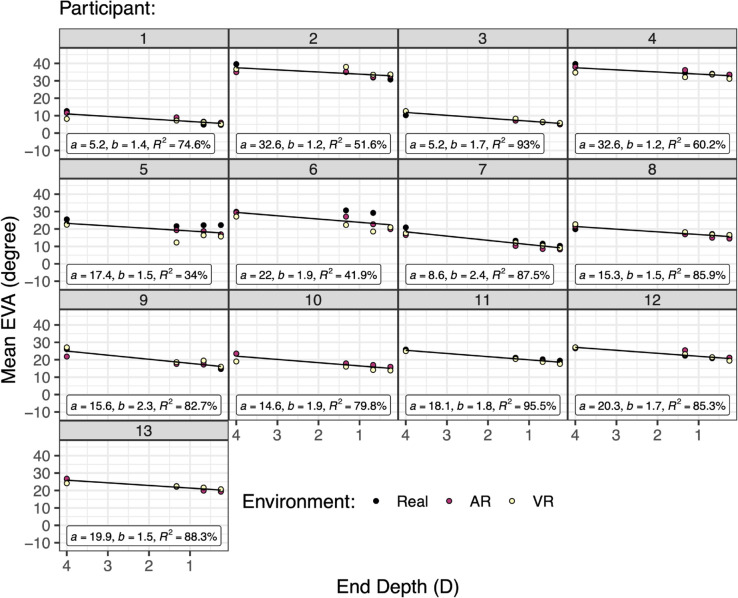
The EVA as predicted by eye movement end depth and participant (data from Fig 4b). The linear model for each participant is added. Note the large variation in intercept (*a*) compared to slope (*b*).

Individual differences were also analyzed. Fig 5 shows the data from Fig 4b separated by *participant*, with a linear model fitted for each participant. Note that participant intercepts ($a:M=17.5^{\circ },\text{SD}=8.6^{\circ })$ have much higher variation than slopes $(b:M=1.7^{\circ /D},\text{SD}=0.37^{\circ /D}$). Because of this, in the mixed-effects models a random intercept term for participant was included, but not a random slope term.

Therefore, to test Hypotheses H1 and H2, a maximal model was created that predicts the measured EVA from the contributions of eye movement end depth and environment:


$${\text{EVA}}_{c}\sim {\text{end depth}}_{c}\;*\;{\text{environment}}_{d}\;+\;(1\;|\;{\text{participant}}_{d}).$$

Models in this paper are expressed in R language format [61,62]. The meaning of this formula is that EVA is predicted by the interaction between end depth and environment, with the addition of an intercept term for each participant. In addition, terms indicated with subscript *c* are *continuous*, adding 1 degree of freedom (DF) to the model, while terms indicated with subscript *d* are *discrete*, adding *N*−1 DFs to the model, where *N* is the number of discrete levels (e.g., 3 for environment). Each DF results in another prediction vector in the final linear equation [63]. Here, *end depth* is a continuous predictor; *environment* is a discrete predictor at the levels of real, AR, and VR; and *participant* is a discrete predictor with a level for each participant.

**Fig 6:**
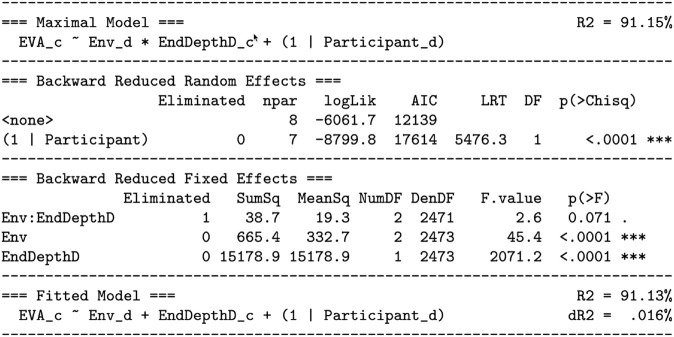
Predicting the eye vergence angle by end depth (Hypothesis H1) and environment (Hypothesis H2): reduction of the maximal model to the fitted model. The reduction uses the technique described by Kuznetsova *et al*. [58]. The *random effects* section tests whether the participant term should be removed, but finds that the amount of described variation would be significantly reduced (p<.0001), and therefore the participant term remains in the fitted model. The *fixed effects* section finds that the Env:EndDepthD interaction term should be removed (p=.071), but that the main effects of ENV and EndDepthD should be retained (p<.0001). For both the maximal and fixed models, the amount of explained variation (*R*^2^) is given, as well as the difference in explained variation between the models (*dR*^2^). The meaning of the columns: Eliminated: If 0, the term is retained; otherwise the order in which a term is eliminated. npar: The number of model parameters; less indicates a better (more parsimonious) model. logLik: The log-likelihood for the model. AIC: The Akaike Information Criterion for the model. Smaller is better. LRT: The likelihood ratio test statistic, which is chi-square distributed. DF: The degrees of freedom for the chi-square test. p(>Chisq): The *p* value for the chi-square test. SumSq, MeanSq, NumDF, DenDF, F.value: The sums of squares, mean square, numerator and denominator degrees of freedom, and *F* statistic of the *F* test. p(>F): The *p* value for the *F* test. This table is constructed using the R functions lme4::lmer, lmerTest::step, and MuMln::r.squaredGLMM. Degrees of freedom are calculated with the Satterhwaite method [58].

As shown in Fig 6, this maximal model was reduced to the fitted model using the step-down model building technique:

$${\text{EVA}}_{c}\sim {\text{end depth}}_{c}\;+\;{\text{environment}}_{d}\;+\;(1\;|\;{\text{participant}}_{d}).$$ (2)

This dropped the end depth:environment interaction term, leaving an additive model that only includes the main effects of end depth and environment. Eq 2 says that *EVA* is predicted by the *end depth* through an estimated slope parameter (because end depth is a continuous predictor), by each level of *environment* through a constant intercept offset (because environment is a discrete predictor), and by each *participant* through a constant intercept offset. Fig 6 also lists *R*^2^, the percentage of variation explained, for both the the maximal model (91.15%) and the fitted model (91.13%), as well as $dR^{2}=\mathrm{.016}\%$, the change in explained variation. The *R*^2^ values are nearly equal, indicating that dropping the interaction term had a very small (and non-significant, *p* = .071) effect on the percentage of variation explained.

**Fig 7:**
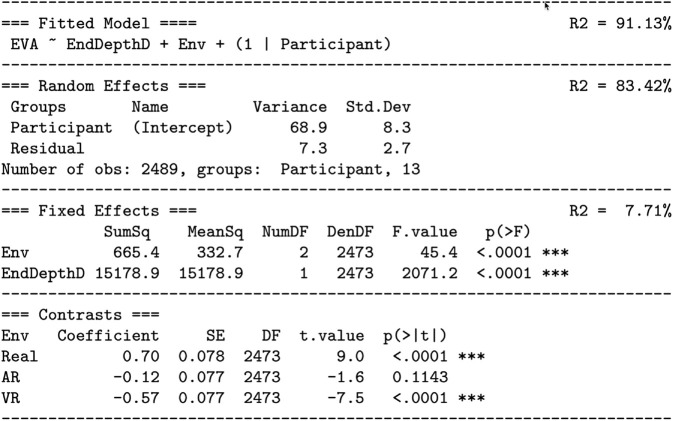
Analysis of Eq 2, the fitted model testing the effect of end depth (Hypothesis H1) and environment (Hypothesis H2) on EVA. The model is visualized in Figs 8 and 9. The *fixed effects* section shows that the effects of both end depth and environment are significant (p<.0001). Overall, the model explains $R^{2}=91.13\%$ of the observed variation in the data, with $R^{2}=83.42\%$ due to the random effects (participant intercept differences), and $R^{2}=7.71\%$ due to the fixed effects (environment and end depth). For each level of environment, Real, AR, and VR, the *contrasts* section gives the distance of each intercept from the overall participant intercept (Fig 9). The contrasts section also tests the significance of each distance. This table is constructed using the R functions lme4::lmer, lmerTest::summary, lmerTest::anova, MuMln::r.squaredGLMM, emmeans::emmeans, and emmeans::contrast. Degrees of freedom are calculated with the Satterhwaite method [58].

**Fig 8:**
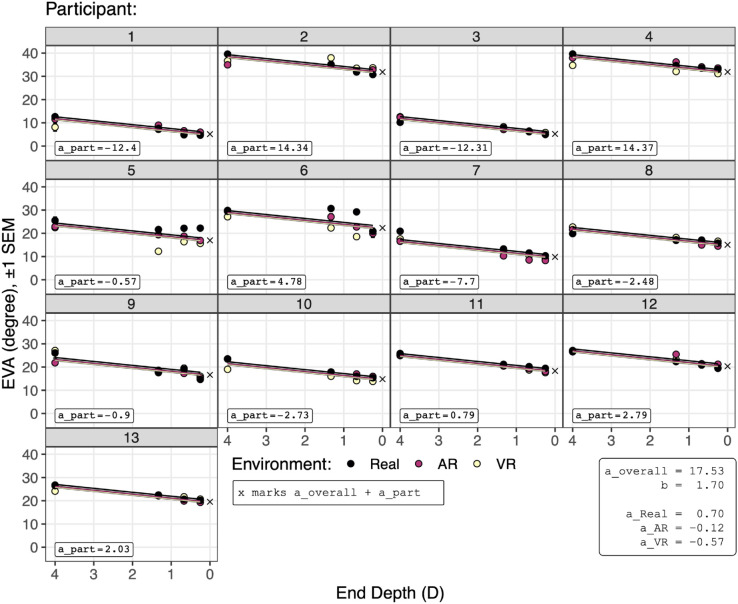
The EVA as predicted Eq 2, the linear mixed effects model. ${\text{EVA}}_{c}\sim {\text{end depth}}_{c}\;+\;{\text{environment}}_{d}\;+\;(1\;|\;{\text{participant}}_{d}).$ The model parameters are shown: the global intercept (a_overall), slope (b), and the intercept offsets for each participant (a_part) and environment (a_Real, a_AR, a_VR). x marks the intercept of each participant, a_overall + a_part. Note the large variation in participant intercepts (a_part) compared to environment intercepts (a_Real, a_AR, a_VR). Also see Fig 9.

**Fig 9:**
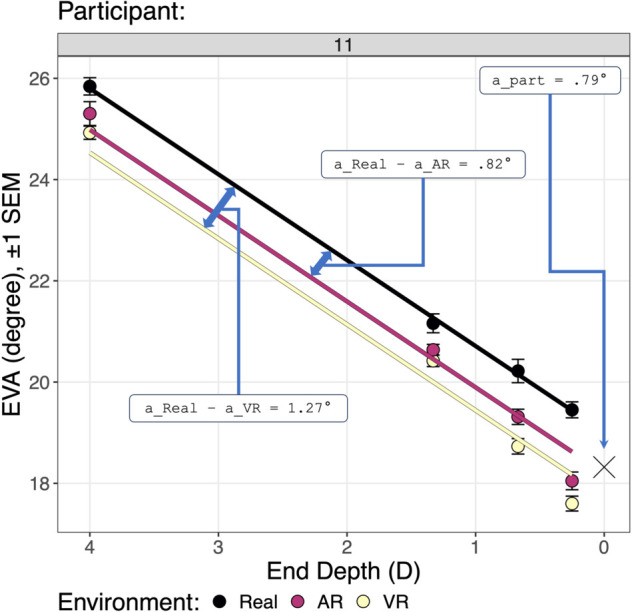
A zoomed-in view of participant 11 from Fig 8. Here, because of the expanded *y* axis, the lines from the fitted model can be seen. This shows the main effect of environment on eye visual angle (EVA): relative to Real targets, EVA was ${\mathrm{.82}}^{\circ }$ smaller for AR targets, and $1.27^{\circ }$ smaller for VR targets. The contrast coefficients (Fig 7) give these distances. x marks a_part = .79, the intercept for participant 11.

Fig 7 analyzes Eq 2, and Figs 8 and 9 visualize Eq 2. Fig 7 also gives the fitted model effect sizes. The overall model explains $R^{2}=91.13\%$ of the variation. This large amount of variation is also visually indicated by the relatively small standard error bars in Figs 8 and 9. Thus, in terms of explanation, the model is quite successful. The table and figures also show that $R^{2}=83.42\%$ of the variation is explained by the random effect of participant intercepts, and $R^{2}=7.71\%$ of the variation is explained by the fixed effects of end depth and environment. This is visually indicated in Fig 8 by the large intercept offsets per participant, especially participants 1 through 4, relative to the overall effect of the end depth and environment.

Indeed, the effect of environment is too small to be clearly seen in Fig 8, given the size and scale of the *y* axis. Fig 9 repeats the view of participant 11 from Fig 8, and through the taller *y* axis more clearly illustrates the effect of both end depth and environment. Fig 9 shows that as the end depth goes from 4 D (.25 meters) to .25 D (4 meters), EVA decreases by $1.70^{\circ }$ per diopter. And, relative to seeing a Real object, EVA decreases by ${\mathrm{.82}}^{\circ }$ when seeing an AR object, and by $1.27^{\circ }$ when seeing a VR object. Note that the line for AR objects is close to participant 11’s intercept (the x symbol, a_part = .79). The *contrasts* section from from Fig 7 shows that the AR offset from x is $-{\mathrm{.12}}^{\circ }$, a nonsignificant difference from participant 11’s viewpoint. However, the VR offset is $-{\mathrm{.57}}^{\circ }$, which does significantly differ (p<.0001), and the Real offset is $+{\mathrm{.70}}^{\circ }$, which also significantly differs (p<.0001). However, these fixed effects only explain 7.71% of the variation (Fig 7). The majority of explained variation, 83.42%, comes from a different eye vergence angle baseline for each participant. Note that the random effects from Fig 7 indicate that among the participant intercepts $\text{SD}=8.3^{\circ }$, a much larger effect than the differences between environments of ${\mathrm{.82}}^{\circ }$ and $1.27^{\circ }$.

Thus, this analysis shows that, for this data, eye vergence angle covaries with the depth of fixated target objects (H1), and this covariation differs according to whether the environment includes real, AR, or VR target objects (H2). Thus, the results are consistent with both hypotheses H1 and H2.

### 4.2 Vergence Stability at End Depth (H3)

We also expected to see evidence of the *vergence stability hypothesis* (H3): when observers fixate a target, the eye vergence angle will be *stable*, regardless of the focal switching depth distance or vergence direction of the eye movement that preceded the fixation. To test this hypothesis, the 2489 EVA measurements were averaged over participant (13), environment (3), and the 12 different combinations of end depth and focal switching distance (Table 1), resulting in 468 conditions. However, as discussed in the section *Data Pre-Processing* above, there were different counts of valid EVA measurements (trials) per condition, and for this analysis, the data is missing three conditions, resulting in 465 analyzed values.

A maximal linear model was created that predicts EVA from the contributions of the focal switching depth of the eye movement, the end depth of the eye movement, and the environment (real, AR, VR) in which the eye movement took place:


$${\text{EVA}}_{c}\sim {\text{switching depth}}_{c}\;*\;{\text{end depth}}_{d}\;*\;{\text{environment}}_{d}\;+\;(1\;|\;{\text{participant}}_{d}).$$

**Fig 10:**
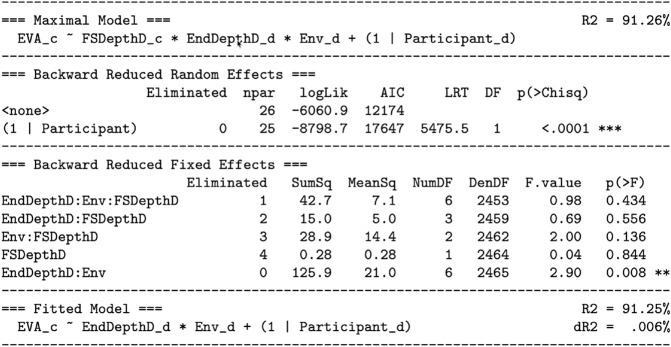
Predicting the vergence stability hypothesis (H3): reducing the maximal model to the fitted model. See the caption for Fig 6.

For this model, because the interest is vergence stability at each tested end depth of the eye movement, end depth is a discrete predictor, at the levels of 4, 1.33, .67, and .25 diopters. As shown in Fig 10, this model was reduced to the fitted model (Eq 3) using the step-down model building technique:

$${\text{EVA}}_{c}\sim {\text{end depth}}_{d}\;*\;{\text{environment}}_{d}\;+\;(1\;|\;{\text{participant}}_{d}).$$ (3)

All interactions with focal switching depth were tested and removed from the model, followed by the main effect of focal switching depth itself. Notable as well is the very small effect size of this removal. Fig 10 lists *R*^2^ for both the maximal model (91.26%) and the fitted model (91.25%). Dropping focal switching depth from the prediction resulted in $dR^{2}=\mathrm{.006}\%$, a very small and non-significant change in explained variation.

**Fig 11:**
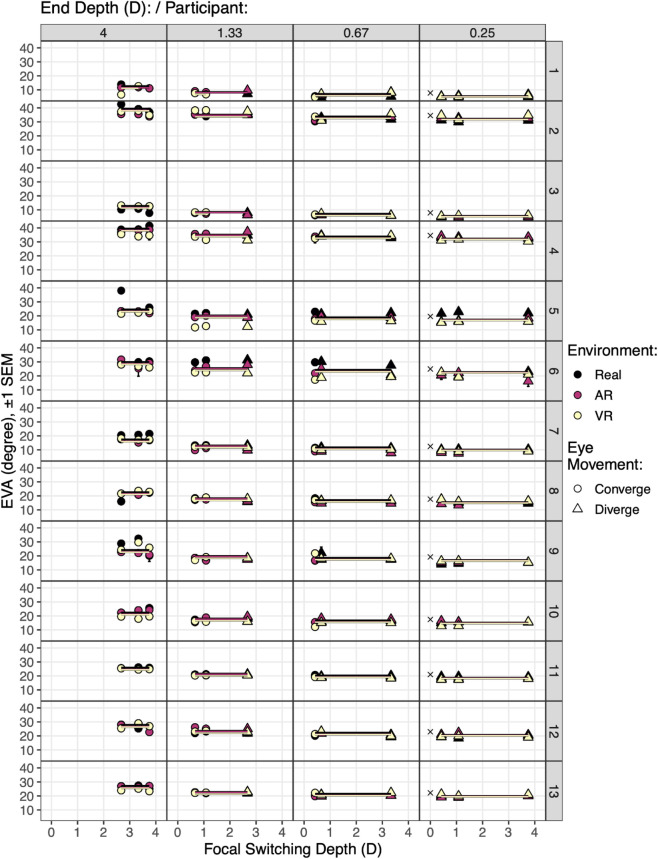
The vergence stability hypothesis (H3), tested with Eq 3: ${\text{EVA}}_{c}\sim {\text{end depth}}_{d}\;*\;{\text{environment}}_{d}\;+\;(1\;|\;{\text{participant}}_{d}).$ The model is illustrated by the lines. Because there are no main effects or interactions with focal switching depth, the lines are horizontal, indicating that when observers fixate a target, the EVA will be stable, regardless of the focal switching depth distance or vergence direction of the eye movement. x marks the intercept of each participant. Also see Fig 12.

**Fig 12:**
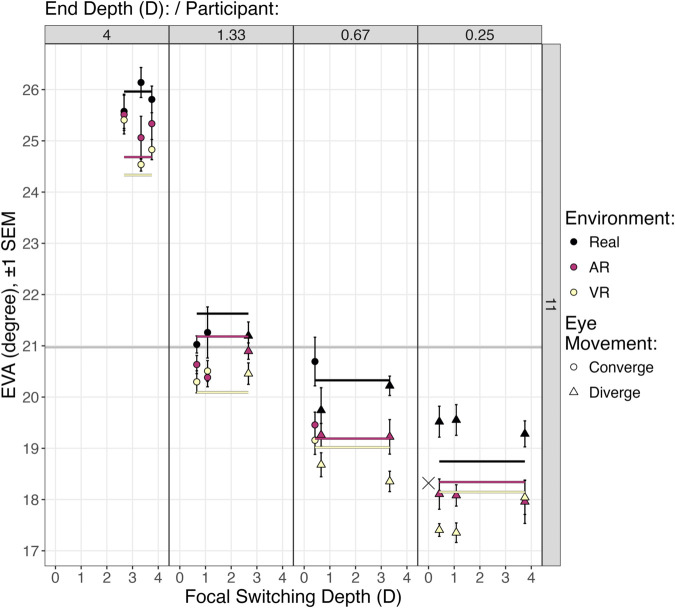
A zoomed-in view of participant 11 from Fig 11. Here, because of the expanded *y* axis, the position of the lines from the fitted model can be seen. The lines represent the levels of environment: Real, AR, and VR. Note that the *y*-position of the lines vary according to the interaction between end depth and environment. The contrast coefficients (Fig 13) give the distance between the grand mean for participant 11 (EVA=20.97) and each line of the fitted model.

Thus, the fitted model suggests that EVA is significantly predicted by the interaction between end depth and environment, with an additive effect of participant. Figs 11 and 12 visualize this model. Because the continuous switching depth predictor fell out of the model, leaving only discrete predictors, when plotted against focal switching depth all of the lines representing Eq 3 are horizontal. Note also that at the closest depth of 4 D (.25 meters), all eye movements converge, and at the farthest depth of .25 D (4 meters), all eye movements diverge. For the two middle distances, some of the eye movements diverge while others converge.

**Fig 13:**
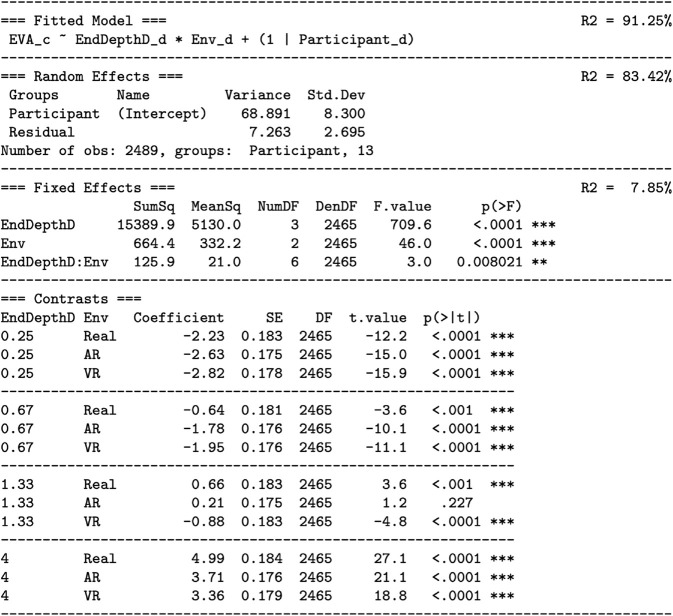
Analysis of Eq 3: the fitted model testing vergence stability (Hypothesis H3). The fitted model is visualized in Figs 11 and 12. See the caption for Fig 7.

Fig 13 analyzes Eq 3. As suggested by the results of the step-down technique, there are significant fixed effects of both end depth, environment, and their interaction. The interaction results are listed in the *contrasts* section, and visualized in Fig 12. There is a strong effect of end depth on EVA, with the largest angle at the closest distance of 4 D (.25 meters). The lines are also ordered according to environment. The interaction shows through the different distances between the lines at each end depth. The contrast coefficients give the distance between the grand mean for participant 11 and each line in Fig 12. Note also that each contrast coefficient significantly differs from this grand mean, with the sole exception of AR targets at 1.33 D. As seen in Fig 12, this line is close enough to the grand mean to not significantly differ (*p* = .227).

Fig 13 also gives the fitted model effect size. Unsurprisingly, this model explains a similar amount of variation as Eq 2, the model that tested H1 and H2 given above. This model (Eq 3) explains $R^{2}=91.25\%$ of the variation, with $R^{2}\;=\;83.42\%$ explained by the random effect of participant intercept, and $R^{2}=7.85\%$ explained by the fixed effect of the interaction between end depth and environment. As discussed above, the dominance of the random effect is also apparent by comparing the standard deviation of participant intercept $\left(8.3^{\circ }\right)$ to the size of the contrast coefficients, which vary from ${\mathrm{.21}}^{\circ }\text{to}4.99^{\circ }$.

Thus, this analysis shows that, for this data, when observers fixate a target, the eye vergence angle is stable, regardless of the focal switching depth distance or vergence direction of the eye movement that preceded the fixation. This analysis is thus consistent with the vergence stability hypothesis (H3).

### 4.3 EVA and Subjective Depth Judgments (H4)

Finally, we hypothesized that subjective depth judgments would be underestimated, and that EVA would provide a more veridical depth estimate than subjective depth judgments (H4). To begin this analysis, it was first necessary to average both verbal reports and EVA measurements into the same count of measurements. The verbal depth reports, which were given in the unit of measurement preferred by the participant, were converted to meters and then into diopters: subjective depth in diopters  = 1/ verbally reported depth in meters. A total of 13 (participant) * 3 (environment) * 4 (end depth) * 3 (repetition) = 468 verbal reports were collected, and then averaged across repetitions into 156 subjective depth judgments. These judgments were then compared to the 2489 EVA measurements, which were also averaged into 13 (participant) * 3 (environment) * 4 (end depth) = 156 EVA values.

Although the two measures (EVA, subjective depth) are expressed in different units (degrees, diopters) and likely come from different distributions, we expected a high degree of correlation. However, as shown in Fig 5, there is a large variation in between-participant intercepts (*a*), as compared to slopes (*b*). In the mixed-effect linear models, these intercepts are subtracted out as part of the modeling computation that examines the fixed predictors [57]; [64, p. 473]. Therefore, to examine the correlation,


normalized EVA=EVA−per-participant intercept

(the values of *a* in Fig 5) was computed for all 2489 vergence measurements. Here, normalized EVA holds the residual values when the strong effect of per-participant intercept is removed. Because normalized EVA removed most between-subject differences, the correlation between normalized EVA and subjective depth was examined. Table 2 shows the Pearson’s correlation coefficient for each environment. The correlations, *r*, are positive, indicating that increasing normalized EVA is associated with increasing subjective depth in diopters (i.e., decreasing subjective depth in meters), in agreement with the geometry of binocular vision (Fig 1). Examining the correlations as *R*^2^ indicates that normalized EVA explains a reasonable percentage of the variation in subjective depth judgments.

**Table 2:** Correlation between subjective depth (D) and normalized EVA (degrees).

Environment	Correlation *r*	Correlation $R^{2}$
Real	0.624	39.0%
AR	0.762	58.1%
VR	0.506	25.6%

**Fig 14:**
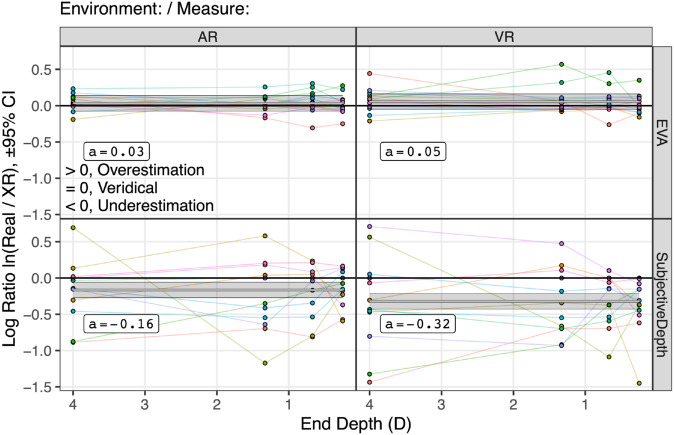
The natural log ratio of Real target depth over XR target depth, plotted as points against the eye movement end depth, environment (AR, VR), and measure (EVA, Subjective Depth). Unlike the previous graphs, which plot means with standard error bars, here all 156 data points are plotted. Participants are different colored points connected by lines. Real target depths are considered veridical, and therefore, for this ratio positive values overestimate veridical depth, while negative values underestimate it. The fitted linear model from Eq 4 is also shown: ${\text{log ratio}}_{c}\;\sim \;{\text{environment}}_{d}\;*\;{\text{measure}}_{d}\;+\;(1\;|\;{\text{participant}}_{d}),$ as gray lines surrounded by ±95% confidence intervals. According to this model, subjective depth judgments were underestimated (see the *contrasts* section of Fig 16), consistent with prior findings. However, EVA measurements were much closer to unity, showing a small and nonsignificant degree of overestimation.

Next, we sought a way to directly compare subjective depth and EVA measurements. As shown in Fig 14, for each participant we calculated the natural log ratio ln(Real/XR) for both EVA and subjective depth measurements. This ratio considers responses to real targets to represent ground truth, and effectively represents both EVA and subjective depth on the same unitless ratio scale. Here,

- a *y*– axis value near 0 indicates a veridical response with respect to the depth of the real target (Real ≈ XR),
- values >0 indicate overestimated XR depths (Real>XR),
- values <0 indicate underestimated XR depths (Real<XR).

The natural logarithm transform normalizes the distribution of ratios to be symmetric around 0 and is more suitable for standard statistical analyses [65]. Fig 14 plots all 156 data points, where the 13 participants are indicated by colored points connected by colored lines.

A maximal linear model was created, which predicts the log ratio by eye movement end depth, environment (AR, VR), and measure (EVA, subjective depth):


$${\text{log ratio}}_{c}\;\sim \;{\text{end depth}}_{c}\;*\;{\text{environment}}_{d}\;*\;{\text{measure}}_{d}\;+\;(1\;|\;{\text{participant}}_{d}).$$

**Fig 15:**
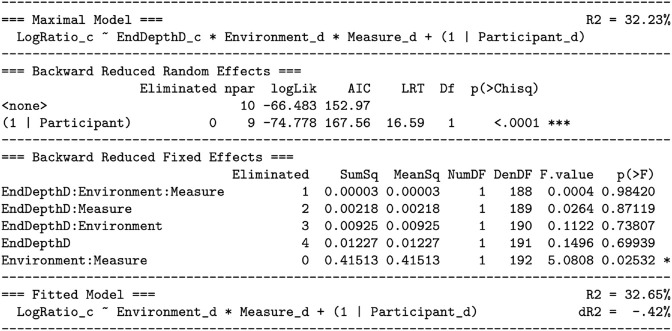
Predicting the natural log ratio of XR target depth over real target depth (Hypothesis H4): reducing the maximal model to the fitted model. See the caption for Fig 6.

As shown in Fig 15, this model was reduced to the fitted model (Eq 4) using the step-down model building technique:

$${\text{log ratio}}_{c}\;\sim \;{\text{environment}}_{d}\;*\;{\text{measure}}_{d}\;+\;(1\;|\;{\text{participant}}_{d}).$$ (4)

Here, all interactions with end depth were tested and removed from the model, followed by the main effect of end depth itself. Notable as well is the very small effect size of this removal. Fig 15 lists *R*^2^ for both the the maximal model (32.23%) and the fitted model (32.65%). Dropping end depth from the prediction resulted in $dR^{2}=-\mathrm{.42}\%$. Not only is this change very small and non-significant, it is in the wrong direction: for the fitted model *R*^2^ went up slightly.

Thus, the fitted model suggests that the log ratio is significantly predicted by the interaction between environment and measure, with an additive effect of participant. Fig 14 visualizes this model, as horizontal lines on top of the data points.

**Fig 16:**
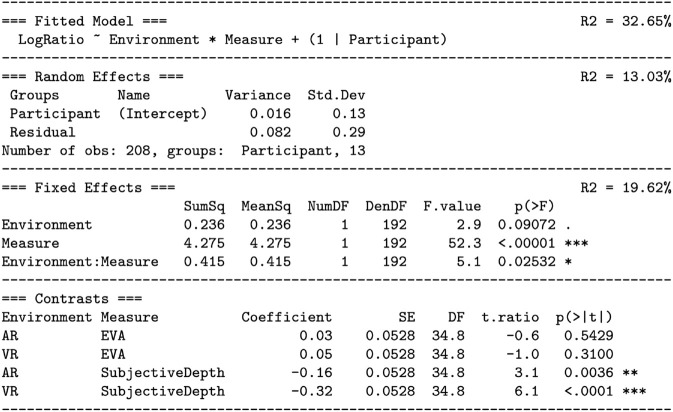
Analysis of Eq 4: the fitted model testing whether subjective depth judgments would be underestimated, and that EVA would provide a more veridical depth estimate than subjective depth judgments (Hypothesis H4). The fitted model is visualized in Fig 14. See the caption for Fig 7.

Fig 16 analyzes Eq 4. As suggested by the results of the step-down technique, there are significant fixed effects of the interaction between environment and measure, and the main effect of measure. Because there is no effect of eye movement end depth, in Fig 14 the model lines are horizontal. The interaction is indicated by a different intercept *a* in each panel. The subjective depth judgments were significantly underestimated (see the *contrasts* section of Fig 16), while the EVA measurements were much closer to unity, showing a small and nonsignificant degree of overestimation.

As shown in Fig 16, the overall model explains $R^{2}=32.65\%$ of the variation. Of this, $R^{2}=19.62\%$ is due to the fixed effect of the environment by measure interaction, and $R^{2}=13.03\%$ is due to the random effect of participant intercepts. Note that, unlike the previous graphs in this paper, this graph shows every data point, and therefore the variation is directly visible. The variation in participant intercept is visualized by the 95% confidence interval around each model line.

This analysis and fitted model shows that, consistent with Hypothesis H4, subjective depth judgments were underestimated. Turning the log ratios back into ratios, AR targets were underestimated by a factor of 1 − $e^{-0.16}=15\%$, and VR targets by a factor of 1 − $e^{-0.32}=27\%$. Also consistent with H4, EVA provides a more veridical depth estimate than subjective depth judgments. The EVA response to AR targets was overestimated by a factor of just *e*^0.03^ − 1=3%, and VR targets by a factor of *e*^0.05^ − 1=5%, and these effects did not significantly differ from 0%. As suggested by the horizontal lines, these effects were constant across the tested distances.

## 5 Discussion

The main goal of this research was to measure eye vergence angle (EVA) from an eye tracker mounted on an optical see-through head-mounted display, and investigate how EVA varied in real, AR, and VR environments for both real and virtual objects placed at different depth locations from the observer. The real, AR, and VR environments were carefully calibrated to be equal in terms of subtended visual angle, location, stereo disparity, and egocentric depth. Furthermore, responses to all environments were collected from each participant, as part of the same experimental session. The research also examined how EVA mapped onto subjective verbal reports of perceived depth.

Participants donned an eye tracker and Microsoft HoloLens 2 while fixating on real, AR, and VR targets located at four different depth positions (0.25, 0.75, 1.5, and 4 meters). We hypothesized that EVA would co-vary with the fixated depth (H1), but this covariation would vary by environment (H2). We also hypothesized that EVA would be stable at the fixated depth regardless of the amount of the focal switching depth and eye movement direction (H3). With regards to subjective depth measurements, we hypothesized that subjective verbal reports would underestimate the depth of XR objects by comparison to real objects, and that objective EVA measurements would provide a more veridical estimate of depth (H4).

### 5.1 End depth (H1)

Our first hypothesis (H1) stated that vergence angle would co-vary with fixed target depth. EVA was found to be consistent with the geometry of perceived depth (Fig 1): EVA was larger for near targets and smaller for far targets, and this pattern followed the expected non-linear relationship that relates vergence angle to depth (Fig 4a) as reported in previous studies [4,29]. By performing a non-linear transform of distance and converting it to diopters (1/distance), we observed a strong linear relationship between EVA and target depth in diopters (Fig 4b). This transformation of units to diopters allowed us to employ standard statistical modeling approaches that assume such linear relationships. Our results strongly support H1 and the validity of our algorithm for accurately measuring EVA from eye tracking data using the Pupil Labs Pupil Core device.

An interesting and unexpected finding was a strong per-participant EVA bias that was constant across the range of target distances. In the experiment, this bias took on the form of a per-participant intercept (Fig 5). This raises an important question: *Which factors contributed to this large bias?*

One possible explanation for the per-participant bias is that it could result from variation in the alignment of the eye tracking cameras with the eyes. Anecdotal evidence for this explanation is that the experimenters noticed that, when measuring their own EVA, repeated measurements that involved removing and then putting the AR display back on their heads and recalibration resulted in baseline shifts demonstrating similarly large differences in intercept (but not slope). For a given observer, each time the AR display is placed back on the head, the calibration, position, and orientation of the eye tracking cameras relative to the eyes changes, which could result in this kind of constant EVA bias that shows up as an intercept. Although this explanation was not examined systematically here, similar effects have been found in AR calibration studies, which are also sensitive to small changes in the way that an AR display sits on the head [66].

This suggests a useful future experiment, in which a participant systematically removes and dons the headset while collecting similar EVA measurements. Furthermore, in our study, we mounted a Pupil Labs Pupil Core eye tracker on a Microsoft HoloLens 2, because at the time the Microsoft eye tracking API did not provide separate gaze vectors for the left and right eyes. However, Microsoft recently added this capability to their eye tracking API, so a future study could also replicate the experiment with the HoloLens 2 integrated eye tracking cameras. This future study might expect a reduction in the per-participant bias.

Another possible explanation for the per-participant bias is interpupillary distance, where larger distances result in larger vergence angles, and smaller distances result in smaller vergence angles. To test this idea, the interpupillary distances of the 13 participants were regressed against the intercepts shown in Fig 5:


intercept~interpupillary distance.

This regression did not predict intercept ($R^{2}=1.4\%$, *F*_1,11_<1), and so the current experiment did not find evidence for this explanation. However, an important caveat is that 13 participants, each contributing a single data point, is too small of a sample size to disprove this hypothesis. In addition, interpupillary distance was measured at infinity, not at the distances tested in the experiment. Therefore, a future study could replicate the experimental procedure while measuring interpupillary distance at each target distance, and again examine any per-participant bias in EVA.

### 5.2 Environment (H2)

Our second hypothesis (H2) was that vergence angle would differ according to whether targets were in a real, AR, or VR environment. H2 was supported (Fig 9), but the effect of the environment was relatively small: EVA measurements for AR targets were ${\mathrm{.82}}^{\circ }$ smaller than for real targets, and VR targets were $1.27^{\circ }$ smaller than for real targets. In terms of the variance explained, the combination of environment and end depth explained only 7.71%, compared to 83.42% for the per-participant intercepts (Fig 7). In addition, the model did not find any interaction effects involving environment, meaning the effect was constant across the tested ranges of the target depth and focal switching depth.

Whether or not this environmental effect is of practical importance will depend upon the application, and the degree to which EVA predicts perceptual performance. The effect could be significant for tasks performed at reaching distances. The experiment showed that a change in EVA as large as $1.27^{\circ }$ (Fig 9) could result in responses to XR targets that were overestimated between 3% (*AR*) to 4.9% (VR) (Fig 14). For a numerical example, at a reaching distance of 40 cm, these could result in a depth change of 1.2 cm (AR) to 2 cm (VR). Compare this to the recommendation from Edwards *et al*. [67], who from clinical experiments suggested that for AR to be useful in image-guided brain surgery, depth error tolerances of 1 mm or less would be required. On the other hand, for tasks performed at action space distances of about 1.5 to 30 meters [68], or for tasks where EVA is not strongly related to perceptual performance, the environmental effect may be less practically important.

One possible explanation for this environmental effect on EVA is the vergence accommodation conflict (VAC). Previous research has observed the after-effect of the VAC on the dynamics of the vergence mechanism [69]. The VAC also causes the vergence angle to bias toward the depth of a display’s fixed focal plane, which can bias perceived depth [70]. Our experiment used the Microsoft HoloLens 2, which has a fixed focal plane at a depth of approximately 1.5 meters (0.67 D). As the real target objects were displayed on monitors, those measured EVA results were not affected by the VAC. However, AR and VR targets should be affected. For the AR and VR targets at .25 and .75 meters (4 and 1.33 D), located in front of the fixed focal plane, EVA values should be biased by the VAC to be smaller than they otherwise would be, corresponding to depths that are farther than they otherwise would be. Fig 9 supports this hypothesis, where at 4 and 1.33 diopters EVA values for AR and VR targets are smaller than real targets. However, this hypothesis also predicts that for the AR and VR targets at 4 meters (.25 D), located behind the fixed focal plane, EVA values should be biased to be larger than they otherwise would be, corresponding to depths that are closer than they otherwise would be. Fig 9 does not support this hypothesis, which found the EVA bias to be constant over all tested distances. Therefore, while the experiment found that VAC could explain some of the effect of environment, it could not explain all of it.

The brightness of an object is a perceptual attribute that is driven by a combination of object luminance and the contrast between the object and its background. Although in the current study luminance and brightness were not measured, it is likely that participants experienced the VR targets as brightest, followed by the AR targets, and then experienced the real targets as dimmest. This is because the VR targets were rendered by a near-eye display against a completely black background, the AR targets were rendered by the same display against a lit background, and the real targets were rendered on monitors positioned in the background. Huckauf [71] investigated how brightness during eye tracker calibration and brightness during a task affect EVA change for a single depth at 63 cm. When they performed the calibration on a bright background and considered the task on a dark background, the vergence angle became smaller and moved further away from the observer. In our experiment, the eye tracker calibration was conducted on a bright background (the Pupil Labs screen-based calibration), but the experimental stimuli were presented on a dark background, with the maximum possible contrast for the VR targets. Similar to the findings of Huckauf [71], the largest EVA was found for the real targets, followed by the AR targets, and then the VR targets had the smallest EVA. Therefore, brightness and contrast effects described by Huckauf [71] are consistent with the environment effects found here.

This possible reason suggests a future study in which EVA is again measured in real, AR, and VR environments, while luminance is measured and systematically manipulated, along with a perceptual measure of perceived brightness. The future study would be further enriched by also measuring perceived depth using a technique such as disparity matching (e.g., Singh *et al*. [8]). Such an experiment could examine how EVA behaves in the presence of perceived depth in many conditions and address many unanswered questions about the perception of virtual and real objects.

### 5.3 Vergence stability (H3)

We hypothesized that vergence angle would be stable regardless of the focal switching depth and vergence direction (H3). We tested this hypothesis in real, AR, and VR environments (Fig 11). In this analysis, the vergence direction of the eye movement was not an independent variable, but varied according to focal switching depth. All eye movements converged for the closest distance of 4 D (.25 meters), while all eye movements diverged for the farthest distance of .25 D (4 meters). Depending on the location of the prior distance, eye movements could converge or diverge at the intermediate distances of 1.33 and .67 D (.75 and 1.5 meters). The linear modeling (Fig 13) shows a strong effect of per-participant intercept (83.42% of explained variation), and a smaller but detectable effect of end depth and environment (7.85%). In total, the linear modeling is very effective, explaining 91.25% of the variation. Notable is the very small effect size of switching depth: .006% of explained variation (Fig 10). Taken together, these effect sizes are statistical evidence for the vergence stability hypothesis (H3). The results indicate that EVA does not specifically depend on the start depth and vergence direction: no matter the size of the vergence eye movement, a stable vergence angle associated with the target’s depth location was measured. A possible reason for this finding is the vergence system’s components in response to depth changes. The vergence system can quickly detect the rapid depth change with the motor signal (the fast vergence component) and can receive continuous neurological feedback for vergence correction through the oculomotor response (the slow feedback component) [69,72].

### 5.4 Subjective depth judgments (H4)

We used subjective measurement in the form of verbal reports to obtain participants’ perceptual experience of depth for each target location. We hypothesized (H4) that subjective depth judgments would show depth underestimation for virtual targets, consistent with prior research [30,39,40], and that objective measurements of eye vergence angle would provide a more veridical estimate of depth (neither underestimation or overestimation). The results supported this hypothesis. As shown in Fig 14, we observed a significant amount of underestimation from subjective depth reports for virtual target depth in AR and VR environments compared to real targets. This effect of underestimation was constant across depth locations and was largest for VR objects (27%) compared to AR objects (15%). We observed a slight overestimation for objective EVA depth measures in AR (3%) and VR (5%), but this differential was much closer to the veridical depth of real targets compared to subjective reports, and was not statistically different than 0% (Fig 16).

Critically, these results suggest that the oculomotor system of users in AR and VR produces vergence eye movements that nearly reflect the veridical distance of virtual targets, as evidenced by EVA measurements that were nearly identical to the vergence eye movements induced by real targets at the same distances. This result casts new light on much previous work that reported that subjective measurements of perceived depth tend to underestimate the actual depth compared to real targets [30,39,40]. Our work suggests that the vergence system itself is likely not the cause of this subjective bias; thus, the tendency for subjective depth underestimation for virtual targets must arise from downstream effects such as cognitive biases in reporting or perceptual illusory biases introduced at a later stage of visual information processing. While our study was not designed to elucidate the nature of such downstream effects on subjective depth perception (whether perceptual, cognitive, or both), future studies could be designed with the aim of disentangling these factors. We believe that such future studies would benefit from simultaneous use of eye tracking to objectively measure EVA to help establish a ground truth for the depth of fixated objects during the task and rule out eye vergence behavior as a potential explanation for observed effects on subjective reports of perceptual depth. Therefore, a potential future study should consider a definite distance perception mechanism (e.g., Bingham & Pagano [73]), including both verbal reports and depth matching between real and virtual objects while measuring EVA.

## 6 Conclusion

This research reports one of the first experiments to measure eye vergence angle (EVA) systematically in real and carefully calibrated extended reality (XR) environments, considering four different depth locations: .25, .75, 1.5, and 4 meters (4, 1.33, .67, .25 diopters). Overall, the main findings are:

**EVA, fixated depth, individual differences:** EVA was strongly associated with the fixated depth in the expected non-linear pattern that relates vergence angle to depth. By performing a non-linear transform of distance and converting it to diopters (1/distance), a strong linear relationship was observed between EVA and target depth in diopters. However, large individual variations in EVA were observed, which may arise from eye tracking calibration, eye camera position, interpupillary distance, brightness and contrast effects, or other unknown factors.

**EVA and XR environment:** EVA differed according to whether the targets were in a real, augmented reality (AR), or virtual reality (VR) environment. The detected differences were small: EVA measurements for AR targets were ${\mathrm{.82}}^{\circ }$ smaller than for real targets, and VR targets were $1.27^{\circ }$ smaller than for real targets. Whether or not these environmental effects will be practically important will depend upon the application, and the unknown degree to which EVA predicts perceptual performance. The environment effects may arise from the vergence accommodation conflict, brightness and contrast effects, or other unknown factors.

**Vergence stability:** Measured EVA was stable with respect to the starting depth of the previously fixated target and invariant to the direction of the vergence eye movement (convergence, divergence). This implies that there is an unbiased one-to-one mapping between EVA and the depth of real and virtual targets.

**EVA and subjective depth:** Subjective depth from verbal reports underestimated the depth of XR targets compared to real targets, while EVA showed very little bias for XR targets compared to real targets. Therefore, a novel finding from this research is that EVA provided a much more veridical estimate of AR and VR target depth, compared to subjective judgment.

**EVA and adaptive XR:** Recently, to provide improved and automated calibration, many head-mounted XR displays (e.g., Microsoft HoloLens 2, Magic Leap 2, HTV Vive Pro Eye, and others) have included built-in eye tracking. In addition to calibration, eye tracking could enable novel ways of interacting with XR content, such as systems that adapt to an observer’s moment-to-moment eye vergence angle. The findings of this experiment suggest that it will be possible to use measured EVA to drive adaptive XR interfaces that change their behavior depending on the user’s vergence angle and verged depth. In addition, such systems will allow investigating novel research questions about how the human visual system operates when engaged by virtual objects at different depths.

## References

[1] Billinghurst M, Clark A, Lee G. A survey of augmented reality. FNT in Human–Computer Interaction. 2015;8(2–3):73–272. doi: 10.1561/1100000049

[2] Van Krevelen DWF, Poelman R. A survey of augmented reality technologies, applications and limitations. IJVR. 2010;9(2):1–20. doi: 10.20870/ijvr.2010.9.2.2767

[3] Gross H, Blechinger F, Achtner B. Handbook of optical systems. 1st ed. Weinheim: Wiley-VCH; 2014.

[4] Arefin MS, Swan II JE, Cohen Hoffing RA, Thurman SM. Estimating perceptual depth changes with eye vergence and interpupillary distance using an eye tracker in virtual reality. In: 2022 Symposium on Eye Tracking Research and Applications. 2022. p. 1–7. 10.1145/3517031.3529632

[5] Steinicke F, Bruder G, Hinrichs K, Kuhl S, Lappe M, Willemsen P. Judgment of natural perspective projections in head-mounted display environments. In: Proceedings of the 16th ACM Symposium on Virtual Reality Software and Technology. 2009. p. 35–42. 10.1145/1643928.1643940

[6] Arefin MS, Phillips N, Plopski A, Gabbard JL, Swan JE. Impact of AR display context switching and focal distance switching on human performance: replication on an AR haploscope. In: 2020 IEEE Conference on Virtual Reality and 3D User Interfaces Abstracts and Workshops (VRW). 2020. 10.1109/vrw50115.2020.00137

[7] Balaban CD, Kiderman A, Szczupak M, Ashmore RC, Hoffer ME. Patterns of pupillary activity during binocular disparity resolution. Front Neurol. 2018;9:990. doi: 10.3389/fneur.2018.00990 30534109 PMC6276540

[8] Singh G, Ellis SR, Swan JE. The effect of focal distance, age, and brightness on near-field augmented reality depth matching. IEEE Trans Vis Comput Graph. 2020;26(2):1385–98. doi: 10.1109/TVCG.2018.2869729 30222576

[9] Cholewiak SA, Love GD, Banks MS. Creating correct blur and its effect on accommodation. J Vis. 2018;18(9):1. doi: 10.1167/18.9.1 30193343 PMC6126933

[10] Krishnan VV, Stark L. A heuristic model for the human vergence eye movement system. IEEE Trans Biomed Eng. 1977;24(1):44–9. doi: 10.1109/TBME.1977.326207 832888

[11] Hoffman DM, Girshick AR, Akeley K, Banks MS. Vergence-accommodation conflicts hinder visual performance and cause visual fatigue. J Vis. 2008;8(3):33.1-30. doi: 10.1167/8.3.33 18484839 PMC2879326

[12] MacKenzie KJ, Hoffman DM, Watt SJ. Accommodation to multiple-focal-plane displays: implications for improving stereoscopic displays and for accommodation control. J Vis. 2010;10(8):22. doi: 10.1167/10.8.22 20884597

[13] Myers GA, Stark L. Topology of the near response triad. Ophthalmic Physiol Opt. 1990;10(2):175–81. doi: 10.1111/j.1475-1313.1990.tb00972.x 2371063

[14] Mon-Williams M, Wann JP. Binocular virtual reality displays: when problems do and don’t occur. Hum Factors. 1998;40(1):42–9. doi: 10.1518/001872098779480622

[15] Wann JP, Rushton S, Mon-Williams M. Natural problems for stereoscopic depth perception in virtual environments. Vision Res. 1995;35(19):2731–6. doi: 10.1016/0042-6989(95)00018-u 7483313

[16] Spiegel DP, Erkelens IM. Vergence-accommodation conflict increases time to focus in augmented reality. J Soc Info Display. 2024;32(5):194–205. doi: 10.1002/jsid.1283

[17] Kramida G. Resolving the vergence-accommodation conflict in head-mounted displays. IEEE Trans Vis Comput Graph. 2016;22(7):1912–31. doi: 10.1109/TVCG.2015.2473855 26336129

[18] Feil M, Moser B, Abegg M. The interaction of pupil response with the vergence system. Graefes Arch Clin Exp Ophthalmol. 2017;255(11):2247–53. doi: 10.1007/s00417-017-3770-2 28815298

[19] Hooge ITC, Hessels RS, Nyström M. Do pupil-based binocular video eye trackers reliably measure vergence?. Vision Res. 2019;156:1–9. doi: 10.1016/j.visres.2019.01.004 30641092

[20] Solé Puig M, Romeo A, Supèr H. Vergence eye movements during figure-ground perception. Conscious Cogn. 2021;92:103138. doi: 10.1016/j.concog.2021.103138 34022640

[21] Sulutvedt U, Mannix TK, Laeng B. Gaze and the eye pupil adjust to imagined size and distance. Cogn Sci. 2018;42(8):3159–76. doi: 10.1111/cogs.12684 30302788

[22] Yaramothu C, Santos EM, Alvarez TL. Effects of visual distractors on vergence eye movements. J Vis. 2018;18(6):2. doi: 10.1167/18.6.2 30029212 PMC5987826

[23] Krauze L, Panke K, Krumina G, Pladere T. Comparative analysis of physiological vergence angle calculations from objective measurements of gaze position. Sensors (Basel). 2024;24(24):8198. doi: 10.3390/s24248198 39771937 PMC11678997

[24] Duchowski AT, Krejtz K, Volonte M, Hughes CJ, Brescia-Zapata M, Orero P. 3D gaze in virtual reality: vergence, calibration, event detection. Procedia Computer Science. 2022;207:1641–8. doi: 10.1016/j.procs.2022.09.221

[25] Wang Z, Zhao Y, Lu F. Control with vergence eye movement in augmented reality see-through vision. In: 2022 IEEE Conference on Virtual Reality and 3D User Interfaces Abstracts and Workshops (VRW). 2022. p. 548–9. 10.1109/vrw55335.2022.00125

[26] Dunn D. Required accuracy of gaze tracking for varifocal displays. In: 2019 IEEE Conference on Virtual Reality and 3D User Interfaces (VR). 2019. p. 1838–42. 10.1109/vr.2019.8798273

[27] Duchowski AT, Pelfrey B, House DH, Wang R. Measuring gaze depth with an eye tracker during stereoscopic display. In: Proceedings of the ACM SIGGRAPH Symposium on Applied Perception in Graphics and Visualization. 2011. p. 15–22. 10.1145/2077451.2077454

[28] Duchowski AT, House DH, Gestring J, Congdon R, Świrski L, Dodgson NA, et al. Comparing estimated gaze depth in virtual and physical environments. In: Proceedings of the Symposium on Eye Tracking Research and Applications. 2014. p. 103–10. 10.1145/2578153.2578168

[29] Iskander J, Hossny M, Nahavandi S. Using biomechanics to investigate the effect of VR on eye vergence system. Appl Ergon. 2019;81:102883. doi: 10.1016/j.apergo.2019.102883 31422246

[30] Gagnon HC, Rosales CS, Mileris R, Stefanucci JK, Creem-Regehr SH, Bodenheimer RE. Estimating distances in action space in augmented reality. ACM Trans Appl Percept. 2021;18(2):1–16. doi: 10.1145/3449067

[31] Swan JE 2nd, Singh G, Ellis SR. Matching and reaching depth judgments with real and augmented reality targets. IEEE Trans Vis Comput Graph. 2015;21(11):1289–98. doi: 10.1109/TVCG.2015.2459895 26340777

[32] Sahm CS, Creem-Regehr SH, Thompson WB, Willemsen P. Throwing versus walking as indicators of distance perception in similar real and virtual environments. ACM Trans Appl Percept. 2005;2(1):35–45. doi: 10.1145/1048687.1048690

[33] Fukusima SS, Loomis JM, Da Silva JA. Visual perception of egocentric distance as assessed by triangulation. J Exp Psychol Hum Percept Perform. 1997;23(1):86–100. doi: 10.1037//0096-1523.23.1.86 9090148

[34] Phillips N, Khan FA, Arefin MS, Bethel CL, Stetanucci J, Swan JE. A conceptual replication and extension of triangulation by walking for measuring perceived distance through a wall. In: 2022 IEEE International Symposium on Mixed and Augmented Reality Adjunct (ISMAR-Adjunct). 2022. p. 278–82. 10.1109/ismar-adjunct57072.2022.00063

[35] Swan JE 2nd, Jones A, Kolstad E, Livingston MA, Smallman HS. Egocentric depth judgments in optical, see-through augmented reality. IEEE Trans Vis Comput Graph. 2007;13(3):429–42. doi: 10.1109/TVCG.2007.1035 17356211

[36] Livingston MA, Zhuming Ai, Swan JE, Smallman HS. Indoor vs. outdoor depth perception for mobile augmented reality. In: 2009 IEEE Virtual Reality Conference. 2009. 10.1109/vr.2009.4810999

[37] Interrante V, Ries B, Anderson L. Distance perception in immersive virtual environments, revisited. In: IEEE Virtual Reality Conference (VR 2006). p. 3–10. 10.1109/vr.2006.52

[38] Knapp JM, Loomis JM. Limited field of view of head-mounted displays is not the cause of distance underestimation in virtual environments. Presence: Teleoperators & Virtual Environments. 2004;13(5):572–7. doi: 10.1162/1054746042545238

[39] Kelly JW, Cherep LA, Siegel ZD. Perceived space in the HTC vive. ACM Trans Appl Percept. 2017;15(1):1–16. doi: 10.1145/3106155

[40] Creem-Regehr SH, Stefanucci JK, Thompson WB, Nash N, McCardell M. Egocentric distance perception in the Oculus Rift (DK2). In: Proceedings of the ACM SIGGRAPH Symposium on Applied Perception. 2015. p. 47–50. 10.1145/2804408.2804422

[41] Rayner K. Eye movements in reading and information processing: 20 years of research. Psychol Bull. 1998;124(3):372–422. doi: 10.1037/0033-2909.124.3.372 9849112

[42] Chinyere Onyemauche U, Makuochi Nkwo S, Elochukwu Mbanusi C, Queeneth Nwosu N. Towards the use of eye gaze tracking technology: Human Computer Interaction (HCI) research. In: Proceedings of the 3rd African Human-Computer Interaction Conference: Inclusiveness and Empowerment. 2021. p. 151–7. 10.1145/3448696.3448710

[43] Jacob RJK. The use of eye movements in human-computer interaction techniques. ACM Trans Inf Syst. 1991;9(2):152–69. doi: 10.1145/123078.128728

[44] Hutt S, D’Mello SK. Evaluating calibration-free webcam-based eye tracking for gaze-based user modeling. In: Proceedings of the 2022 International Conference on Multimodal Interaction. 2022. p. 224–35. 10.1145/3536221.3556580

[45] Sidenmark L, Clarke C, Newn J, Lystbæ k MN, Pfeuffer K, Gellersen H. Vergence matching: inferring attention to objects in 3D environments for gaze-assisted selection. In: Proceedings of the 2023 CHI Conference on Human Factors in Computing Systems. 2023. p. 1–15. 10.1145/3544548.3580685

[46] Plopski A, Hirzle T, Norouzi N, Qian L, Bruder G, Langlotz T. The eye in extended reality: a survey on gaze interaction and eye tracking in head-worn extended reality. ACM Comput Surv. 2022;55(3):1–39. doi: 10.1145/3491207

[47] Grubert J, Itoh Y, Moser K, Swan JE. A survey of calibration methods for optical see-through head-mounted displays. IEEE Trans Vis Comput Graph. 2018;24(9):2649–62. doi: 10.1109/TVCG.2017.2754257 28961115

[48] Itoh Y, Kunze K, Plopski A, Sandor C. NII Shonan meeting report: augmented reality in human-computer interaction. 2018 –8. 2018. https://shonan.nii.ac.jp/seminars/135/

[49] Arefin MS, Phillips N, Plopski A, Gabbard JL, Swan JE. The effect of context switching, focal switching distance, binocular and monocular viewing, and transient focal blur on human performance in optical see-through augmented reality. IEEE Trans Vis Comput Graph. 2022;28(5):2014–25. doi: 10.1109/TVCG.2022.3150503 35167470

[50] Khan FA, Muvva VVRMKR, Wu D, Arefin MS, Phillips N, Swan JE. Measuring the perceived three-dimensional location of virtual objects in optical see-through augmented reality. In: 2021 IEEE International Symposium on Mixed and Augmented Reality (ISMAR). 2021. p. 109–17. 10.1109/ismar52148.2021.00025

[51] Khan FA, Muvva VVRMKR, Wu D, Arefin MS, Phillips N, Swan JE. A method for measuring the perceived location of virtual content in optical see through augmented reality. In: 2021 IEEE Conference on Virtual Reality and 3D User Interfaces Abstracts and Workshops (VRW). 2021. p. 657–8. 10.1109/vrw52623.2021.00211

[52] Khan FA, Arefin MS, Phillips N, Swan JE. A replication study to measure the perceived three-dimensional location of virtual objects in optical see through augmented reality. In: 2022 IEEE Conference on Virtual Reality and 3D User Interfaces Abstracts and Workshops (VRW). 2022. p. 796–7. 10.1109/vrw55335.2022.00249

[53] Magic Leap Inc. Magic Leap 2 Optics Breakthroughs Empower Enterprise AR. Magic Leap Newsroom. 2024. https://www.magicleap.com/newsroom/magic-leap-2-optics-breakthroughs-empower-enterprise-ar

[54] Miles WR. Ocular dominance in human adults. The Journal of General Psychology. 1930;3(3):412–30. doi: 10.1080/00221309.1930.9918218

[55] Dunn D, Tursun O, Yu H, Didyk P, Myszkowski K, Fuchs H. Stimulating the human visual system beyond real world performance in future augmented reality displays. In: 2020 IEEE International Symposium on Mixed and Augmented Reality (ISMAR). 2020. p. 90–100. 10.1109/ismar50242.2020.00029

[56] Solé Puig M, Pérez Zapata L, Aznar-Casanova JA, Supèr H. A role of eye vergence in covert attention. PLoS One. 2013;8(1):e52955. doi: 10.1371/journal.pone.0052955 23382827 PMC3561361

[57] Bates D, Mächler M, Bolker B, Walker S. Fitting linear mixed-effects models Usinglme4. J Stat Soft. 2015;67(1):1–48.doi: 10.18637/jss.v067.i01

[58] Kuznetsova A, Brockhoff PB, Christensen RHB. lmerTest package: tests in linear mixed effects models. J Stat Soft. 2017;82(13) :1–26..doi: 10.18637/jss.v082.i13

[59] Jaeger BC, Edwards LJ, Das K, Sen PK. AnR2statistic for fixed effects in the generalized linear mixed model. Journal of Applied Statistics. 2016;44(6):1086–105. doi: 10.1080/02664763.2016.1193725

[60] Nakagawa S, Schielzeth H. A general and simple method for obtaining R2 from generalized linear mixed-effects models. Methods Ecol Evol. 2012;4(2):133–42. doi: 10.1111/j.2041-210x.2012.00261.x

[61] Chambers JM, Hastie TJ. Statistical Models in S. Pacific Grove, CA, USA: Chapman & Hall; 1993.

[62] R Core Team. R: A Language and Environment for Statistical Computing; 2023. https://www.R-project.org/

[63] Pedhazur EJ. Multiple regression in behavioral research. 2nd ed. Holt, Rinehart, & Winston. 1982.

[64] Howell DC. Statistical methods for psychology. 5th ed. Wadsworth. 2001.

[65] Bland JM, Altman DG. Transformations, means, and confidence intervals. BMJ. 1996;312(7038):1079. doi: 10.1136/bmj.312.7038.1079 8616417 PMC2350916

[66] Moser K, Itoh Y, Oshima K, Swan JE, Klinker G, Sandor C. Subjective evaluation of a semi-automatic optical see-through head-mounted display calibration technique. IEEE Trans Vis Comput Graph. 2015;21(4):491–500. doi: 10.1109/TVCG.2015.2391856 26357099

[67] Edwards PJ, Johnson LG, Hawkes DJ, Fenlon MR, Strong AJ, Gleeson MJ. Clinical experience and perception in stereo augmented reality surgical navigation. Lecture Notes in Computer Science. Berlin, Heidelberg: Springer; 2004. p. 369–76. 10.1007/978-3-540-28626-4_45

[68] Cutting JE, Vishton PM. Perceiving layout and knowing distances. Perception of Space and Motion. Elsevier; 1995. p. 69–117. 10.1016/b978-012240530-3/50005-5

[69] Vienne C, Sorin L, Blondé L, Huynh-Thu Q, Mamassian P. Effect of the accommodation-vergence conflict on vergence eye movements. Vision Res. 2014;100:124–33. doi: 10.1016/j.visres.2014.04.017 24835799

[70] Kruijff E, Swan JE, Feiner S. Perceptual issues in augmented reality revisited. In: 2010 IEEE International Symposium on Mixed and Augmented Reality. 2010. p. 3–12. 10.1109/ismar.2010.5643530

[71] Huckauf A. Systematic shifts of fixation disparity accompanying brightness changes. In: Proceedings of the 2018 ACM Symposium on Eye Tracking Research & Applications. 2018. p. 1–5. 10.1145/3204493.3204587

[72] Hung GK, Ciuffreda KJ, Rosenfield M. Proximal contribution to a linear static model of accommodation and vergence. Ophthalmic Physiologic Optic. 1996;16(1):31–41. doi: 10.1046/j.1475-1313.1996.95001107.x 8729564

[73] Bingham GP, Pagano CC. The necessity of a perception-action approach to definite distance perception: monocular distance perception to guide reaching. J Exp Psychol Hum Percept Perform. 1998;24(1):145–68. doi: 10.1037//0096-1523.24.1.145 9483825

